# Distinct defects in early innate and late adaptive immune responses typify impaired fracture healing in diet-induced obesity

**DOI:** 10.3389/fimmu.2023.1250309

**Published:** 2023-10-03

**Authors:** Deepak Kumar Khajuria, Irene Reider, Fadia Kamal, Christopher C. Norbury, Reyad A. Elbarbary

**Affiliations:** ^1^ Department of Orthopaedics and Rehabilitation, The Pennsylvania State University College of Medicine, Hershey, PA, United States; ^2^ Center for Orthopaedic Research and Translational Science (CORTS), The Pennsylvania State University College of Medicine, Hershey, PA, United States; ^3^ Department of Microbiology and Immunology, The Pennsylvania State University College of Medicine, Hershey, PA, United States; ^4^ Department of Pharmacology, The Pennsylvania State University College of Medicine, Hershey, PA, United States; ^5^ Department of Biochemistry and Molecular Biology, The Pennsylvania State University College of Medicine, Hershey, PA, United States; ^6^ Center for RNA Molecular Biology, Pennsylvania State University, University Park, State College, PA, United States

**Keywords:** innate, adaptive, immunity, obesity, diabetes, bone, fracture, healing

## Abstract

Bone fractures, the most common musculoskeletal injuries, heal through three main phases: inflammatory, repair, and remodeling. Around 10% of fracture patients suffer from impaired healing that requires surgical intervention, a huge burden on the healthcare system. The rate of impaired healing increases with metabolic diseases such as obesity-associated hyperglycemia/type 2 diabetes (T2D), an increasing concern given the growing incidence of obesity/T2D. Immune cells play pivotal roles in fracture healing, and obesity/T2D is associated with defective immune-cell functions. However, there is a gap in knowledge regarding the stoichiometry of immune cells that populate the callus and how that population changes during different phases of healing. Here, we used complementary global and single-cell techniques to characterize the repertoire of immune cells in the fracture callus and to identify populations specifically enriched in the fracture callus relative to the unfractured bone or bone marrow. Our analyses identified two clear waves of immune-cell infiltration into the callus: the first wave occurs during the early inflammatory phase of fracture healing, while the second takes place during the late repair/early remodeling phase, which is consistent with previous publications. Comprehensive analysis of each wave revealed that innate immune cells were activated during the early inflammatory phase, but in later phases they returned to homeostatic numbers and activation levels. Of the innate immune cells, distinct subsets of activated dendritic cells were particularly enriched in the inflammatory healing hematoma. In contrast to innate cells, lymphocytes, including B and T cells, were enriched and activated in the callus primarily during the late repair phase. The Diet-Induced Obesity (DIO) mouse, an established model of obesity-associated hyperglycemia and insulin resistance, suffers from multiple healing defects. Our data demonstrate that DIO mice exhibit dysregulated innate immune responses during the inflammatory phase, and defects in all lymphocyte compartments during the late repair phase. Taken together, our data characterize, for the first time, immune populations that are enriched/activated in the callus during two distinct phases of fracture healing and identify defects in the healing-associated immune response in DIO mice, which will facilitate future development of immunomodulatory therapeutics for impaired fracture healing.

## Introduction

Bone fracture healing is a complex regenerative process that involve numerous cell types. Most cortical bone fractures heal through endochondral ossification. The healing process proceeds via 3 main overlapping phases: inflammatory, repair, and remodeling (1). During the initial inflammatory phase, immune cells are recruited to the healing hematoma and secrete various cytokines and growth factors that regulate recruitment, proliferation, and differentiation of stem cells and progenitors, setting the stage for the repair phase (1, 2). During the early repair phase, the progenitors differentiate into chondrocytes that initially proliferate to form a dense fibrocartilaginous callus across the fracture gap (1–3). The chondrocytes then undergo hypertrophy and mineralization, which hardens the fracture gap, allowing the formation of new blood vessels across the fracture site (1–3). The fibrocartilaginous callus is then gradually replaced by newly formed woven bone, which ensues the remodeling phase to re-establish the characteristic laminar structure of cortical bone (1–3).

The inflammatory phase is integral to the healing process, and although it is short lived, the impact of immune cells on fracture healing is thought to extend beyond the early phases of healing (2). Recent studies have identified a role for both innate and adaptive immune cell populations in fracture healing. In the innate compartment, neutrophils remove tissue debris during the early phase of healing and secrete cytokines that recruit monocytes to the callus (1, 4–6). Monocytes differentiate into macrophages, which are enriched in the hematoma of day 3 post-fracture (d3) (7) and whose role in fracture healing is still being investigated. Depletion of macrophages during fracture healing results in impaired bone formation and delayed union (8, 9). The function of macrophages in fracture healing depends on whether they are classically activated M1 or alternatively activated M2 macrophages. M1 macrophages secret inflammatory cytokines that attract more monocytes to the fracture callus (10). In contrast, M2 macrophages secrete anti-inflammatory cytokines during the later inflammatory stages that induce angiogenesis and recruit and stimulate the differentiation of mesenchymal progenitors (11–13). In the adaptive immune compartment, roles for both B and T lymphocytes have been reported, although they are far less studied than macrophages. Depletion of B cells or T cells delays fracture healing (14–16). B cells and T cells secrete factors that co-regulate osteoclast differentiation and activity (17–19), and B cells also suppress some pro-inflammatory signals (17). Current studies aim to better understand the roles of the immune system in bone regeneration in an effort to develop immunomodulatory therapeutics that enhance bone fracture healing.

Dysfunction of immune cells due to aging or metabolic diseases results in impaired bone healing, which can be ameliorated by parabiosis or bone marrow (BM) transplantation (20, 21). For example, diabetic patients suffer from higher rates of nonunion (22). Studies in different diabetic mouse models also revealed several abnormalities in the healing callus and delayed fracture healing (22). The Diet-Induced Obesity (DIO) mouse is an established model for obesity and its accompanying hyperglycemia, glucose intolerance, and insulin insensitivity (3). DIO mice exhibit impaired fracture healing, a phenotype that has been attributed to several underlying mechanisms, including defective functions of bone progenitors, premature resorption of the fibrocartilaginous callus, defective bone formation, and abnormal structure of collagen fibers (3, 23, 24). Given the reported roles of immune cells in the recruitment and osteogenic differentiation of bone progenitors (2), it is possible that the defects observed in the fracture healing of DIO mice is, at least in part, the outcome of a defective immune response. Generally, obesity and diabetes are associated with mild systemic inflammation and immune dysfunction (25). However, the impact of systemic immune dysfunction upon the immune response during bone fracture healing in the DIO mice has never been investigated.

Despite the growing evidence of the central roles that the immune system plays in fracture healing, there is dearth of information regarding the composition/stoichiometry of the immune cells in the fracture callus and how that composition changes during different phases of healing. In this study, we use complementary methods, which include global and single-cell analyses, to comprehensively analyze the immune cells that populate the healing callus during the inflammatory phase and the late repair phase of fracture healing. We also compare the composition of immune cells in the callus of lean mice to that of the DIO mice to identify novel targets for immunomodulatory therapeutic agents.

## Results

### The infiltration of immune cells into the fracture callus peaks during the early inflammatory phase and the late repair/remodeling phase

**Figure 1:**
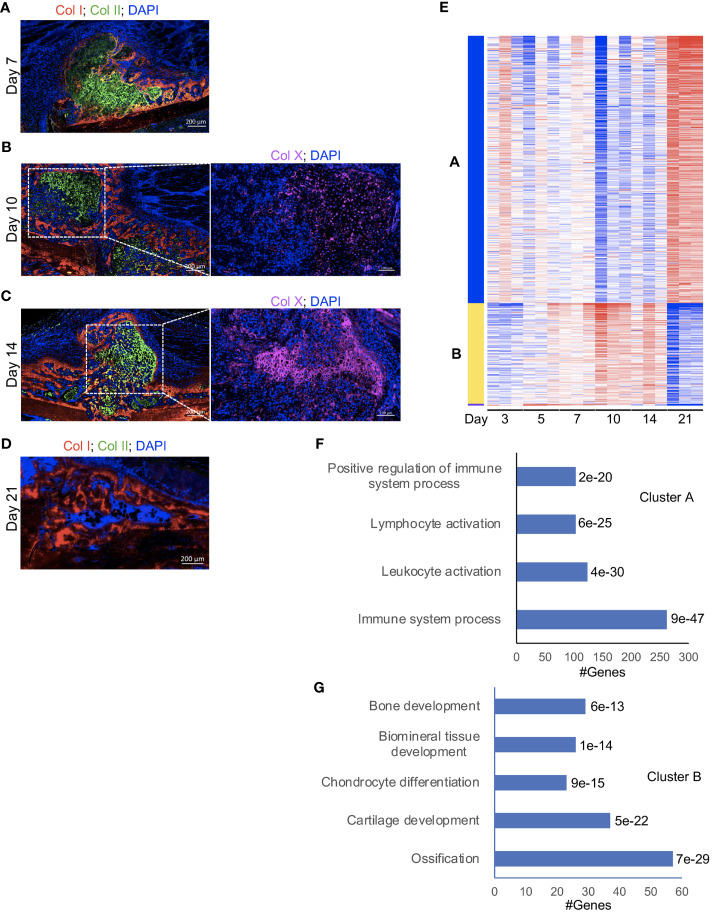
A strong influx of immune cells into the callus during the late phases of fracture healing. (**A-D**, left) IF co-staining of Col I (red) and Col II (green) in callus tissues harvested at the indicated timepoints. Between days 7 and 14 post-fracture, Col II marks the soft-callus chondrocytes, which occupy the fracture gap, while red staining marks the newly formed bone, which occupies callus areas distal to the fracture line. On day 21 post-fracture, Col II (green staining) is absent, indicating resorption of the soft callus, and the fracture gap is completely bridged by the newly formed woven bone. (**B, C**, right) IF staining of the chondrocyte hypertrophy marker Col X (magenta). The images were taken in the soft-callus regions marked by the white boxes on the left panels. DAPI stains nuclei (blue). The scale bar = 200 μm in the left panels and 100 μm in the right panels. All images are representative of 5 callus tissues [see also **Figure S1** and (3)]. **(E)** A heatmap of K-means clustering of the differentially expressed genes on the indicated post-fracture days (see also **Table S1**). Genes were grouped in 2 clusters **(A, B)** based on the pattern of gene expression among different days. **(F, G)** Representative pathways in which genes in cluster A **(F)** and cluster B **(G)** were enriched. The *P_adj_* of enrichment for each pathway is given (see also **Table S6**). RNA-seq was performed on 3 mice at each timepoint.

We used an open tibial mid-diaphyseal murine fracture model, which is an established model for endochondral bone healing (3, 23, 26). We and others have published extensive analysis of the healing time course in this model (3, 23, 26), and select immunofluorescence (IF) staining and histological analyses of the healing callus at different post-fracture timepoints are shown in **Figures 1A-D**; **S1A-D**. On d7 post-fracture in this model, the areas within and immediately surrounding the fracture gap were filled with chondrocytes that formed a dense fibrocartilaginous area (soft callus) (3, 26) (**Figures 1A**, **S1A**). As healing proceeded to days 10 and 14, soft-callus chondrocytes underwent hypertrophic differentiation as indicated by expression of the hypertrophy markers (3, 26) (Collagen 10 [Col X] staining is shown in **Figures 1B, C**). The callus areas distal to the fracture gap were composed of newly formed woven bone (**Figures 1A-C**; **S1A-C**). As healing further progressed to d21, there was complete replacement of the soft callus with woven bone, resulting in bony bridging of the fracture gap and re-establishment of the intramedullary cavity and bone marrow (BM) elements (**Figures 1D**, **S1D**).

In a recently published work (26), we comprehensively analysed days 14 and 21 post-fracture of the same fracture model using RNA-seq, reverse transcriptase- quantitative polymerase chain reaction (RT-qPCR), and IF staining, and we showed a strong influx of immune cells to the healing callus between days 14 and 21. In this current study, we expanded our RNA-seq experiments to include days 3, 5, 7, 10, 14, and 21 to provide an overarching insight into the progression of the immune response as well as the formation of osseous elements during the healing process. Principal Component Analysis (PCA) indicated clustering of the timepoints into 3 main groups: days 3, 5, and 7 in the first group, days 10 and 14 in the second group, and d21 in the third group (**Figure S2**). Analysis of individual days indicated that, as expected, the expression of genes associated with the immune/inflammatory response were readily detectable and highly expressed in the d3 callus (**Tables S1**, **S2**). Progression of the healing response to d5 was accompanied by an increase in the expression of genes associated with cartilage and connective tissue development (**Figures S3A, B**; **Tables S1**, **S2**), but the overall pattern of expression of immune-response-associated genes was comparable to that of d3 (**Figures S3A, B**; **Tables S1**, **S2**). As healing proceeded beyond this initial inflammatory phase to the cartilaginous callus repair phase (days 7 to 14), there was a marked increase in the expression of genes involved in chondrocyte differentiation, cartilage and bone development, and ossification (**Figures S4-S6**; **Tables S3**-**S5**). Consistently, k-Means clustering indicated clustering of genes involved in cartilage and bone development into a group that shows the peak expression between days 7 and 14 (**Figures 1E** “cluster B”, **G**; **Table S6**). These data are consistent with our histological analysis and IF staining (**Figures 1A-D**, **S1A-D**) as well as published data (3, 26). Progression into this cartilaginous callus phase was also accompanied by a marked decrease in the expression of genes associated with immune and inflammatory response, leukocyte migration and activation, and cytokine production (**Figures 1E** “cluster A”, **F**; **S4-S6**; **Tables S3**-**S6**), indicating that the early immune response was partially resolved. Importantly, as we recently reported (26), the expression of genes associated with immune cells and immune response peaked again on d21 (**Figures 1E** “cluster A”, **F**; **S7A, B**; **Tables S6**, **S7**), which was further confirmed by analyzing the expression of the general immune-cell marker Cd45 (**Figure S7C**). This biphasic pattern of immune cell infiltration in the callus is interesting; while the early infiltration wave during the initial inflammatory phase is well established, the observed late infiltration phase is uninvestigated. From these data, it remains unclear whether the observed late increase in immune activity was due to re-establishment of BM elements in the d21 callus (**Figures 1D**; **S1D**), or due to active recruitment of immune cells to the healing callus.

### The repertoire of myeloid cells in the fracture callus, unfractured bone, and bone marrow

Our RNA-seq data indicated that immune infiltration peaked twice in the healing callus: during the initial inflammatory phase and on d21 post-fracture (**Figures 1E, F**; **S3-7**; **Tables S1**-**S7**). However, the principal pathways identified by the RNA-seq data revealed only a high-level view of changes in the immune and inflammatory responses during the healing process. To investigate the detailed cellular composition of the immune cells within the callus at each peak of immune activation, we analyzed the callus during the initial inflammatory phase and on d21 post-fracture using multiparameter flow cytometry (FC). We chose to analyze day 5 during the initial inflammatory phase because we could isolate 5-10 times more total cells, and thus many more CD45^+^ immune cells, from the d5 callus than the d3 callus. Furthermore, the expression of genes associated with general immune and inflammatory response was comparable on days 3 and 5 (**Figures S3A, B**; **Tables S1**, **S2**). We also compared the cells present in the callus at these time points to those in the contralateral unfractured bone (thoroughly washed out to remove BM) or in the BM isolated from the contralateral unfractured bone. In this way we were able to identify changes that were specific to the callus, and to assess whether the immune-cell infiltration we observed on d21 (**Figures 1E, F**; **S7**, **Tables S1**, **S6**, **S7**) was due to re-establishment of the normal BM microenvironment, or to active recruitment of specific immune cells to the callus. Lastly, although the d5 callus consists of an inflammatory hematoma and does not contain bone marrow, we surmised that a comparison of the cellular composition of the d5 callus to that of bone and BM would provide a strong indication of cell types that may play a requisite role in efficient initiation of fracture healing during the initial inflammatory phase.

The overall cellularity of callus vs. bone vs. bone marrow differs widely with the tissue being examined, with the BM being the tissue with the highest cellularity among the three. Therefore, a direct comparison of the cell numbers within individual populations among different tissues is not informative. Rather, we portrayed our results as % of single cells to allow comparison between tissues and to ascertain the proportion and relative enrichment of any individual cell type and, thus, the contribution of individual cell populations to each tissue.

**Figure 2:**
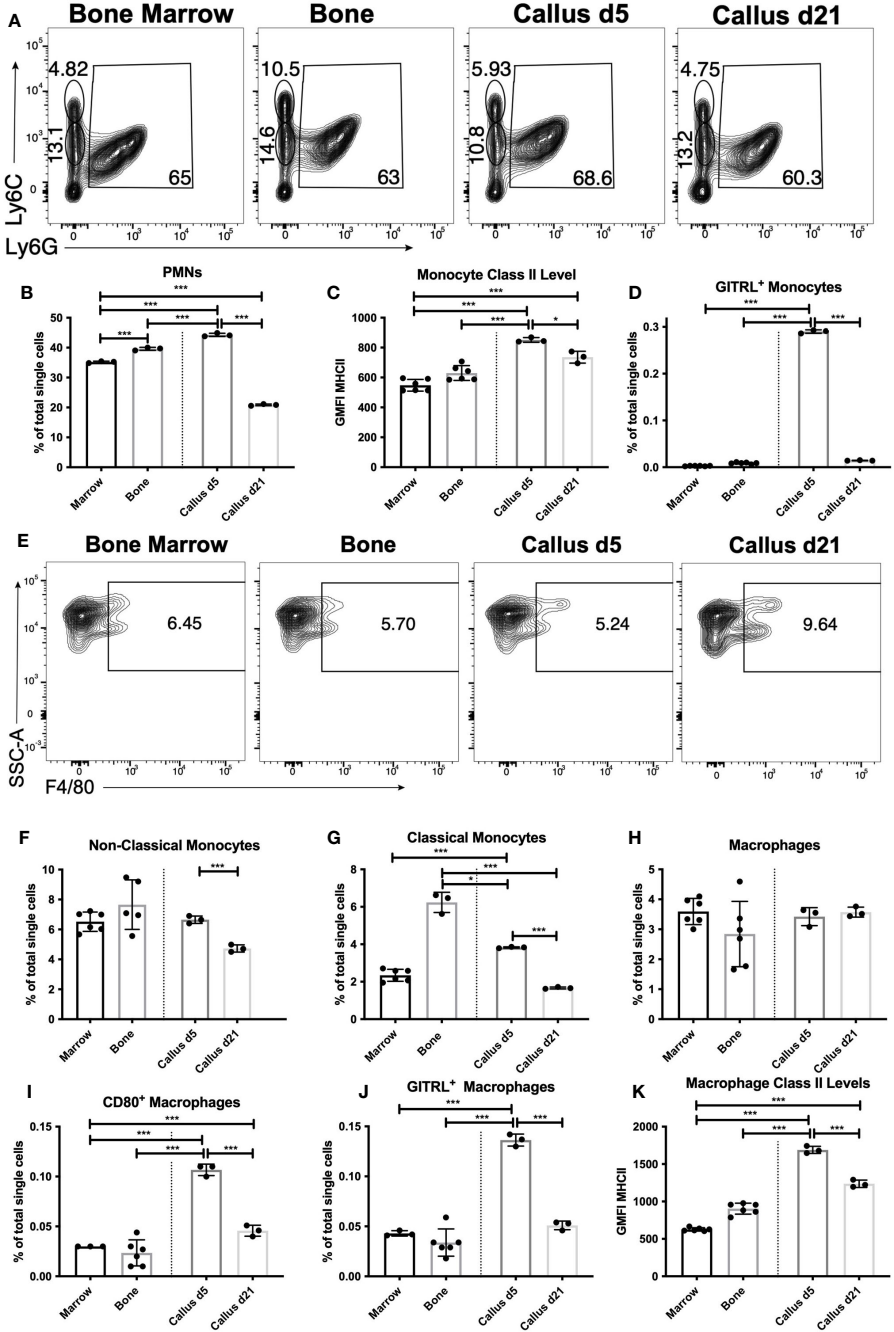
Recruitment and activation of innate immune populations during the early inflammatory phase of healing. Single cell populations liberated from marrow, bone or callus on day 5 (d5) or d21 post-fracture were analyzed by FC using the gating strategies outlined in **Table S8** and shown in **(A)** (PMNs, classical and non-classical monocytes) and **(E)** (macrophages). Quantification of **(B)** PMNs and PMN precursors, **(F)** non-classical monocytes, **(G)** classical monocytes, and **(H)** macrophages as a proportion of total single cells in each population. Activation of myeloid cell populations was assessed by measuring cell surface levels of MHC Class II **(C, K)** or the proportion of singlets expressing the costimulatory molecules GITRL **(D, J)** or CD80 **(I)**. N = 3 for callus tissues (5 calli were pooled in each replicate); N = 3 – 6 for bone and BM. The bar graphs present average ± SEM. (*) *P* < 0.05, and (***) *P* < 0.001 using one-way analysis of variance (ANOVA) followed by Tukey’s *post hoc* test.

Initially we examined the proportion of myeloid cells within d5 or d21 callus vs. bone and BM using FC. The staining panel used in the analysis is shown in **Table S8**. Myeloid cells, such as neutrophils, monocytes and macrophages, are a large constituent of the BM and play well-described roles in fracture healing (2, 5, 27–33). FC analysis distinguishing polymorphonuclear granulocytes (PMNs) from monocytes within an F4/80^-^ population is shown in **Figure 2A**, but PMNs could not be distinguished from myeloid cell precursors using this staining panel. PMNs, potentially including neutrophils, eosinophils and basophils, were the largest single population within bone, BM and the calli on d5 and d21. Unfractured bone contained a slightly higher proportion of PMNs than BM, and this proportion was further increased in the d5 callus (**Figure 2B**). However, by d21 there was a marked reduction in the proportion of PMNs to levels significantly below those found in BM (**Figure 2B**). There was no significant increase in the proportion of bulk monocytes in the callus (either d5 or d21) vs. BM, and an unexpected reduction in both the calli of d5 and d21 compared to the unfractured bone (**Figure S8A**).

We also analyzed the activation of monocytes by measuring the cell surface expression of costimulatory molecules, including CD86, GITRL, and cell surface major histocompatibility complex (MHC) Class II. CD86 levels in bulk monocytes were higher in bone than in BM, with levels in the callus of d5 sitting in between the two, and levels in the callus of d21 comparable to those in BM (**Figure S8B**). The number of MHC Class II-expressing monocytes were similar in all tissues (**Figure S8C**), but the cell surface levels of MHC II were higher in the monocytes of d5 callus than in either bone or BM (**Figure 2C**), indicating activation of some monocytes in the d5 callus. This was further confirmed by a marked upregulation in the expression of GITRL in a small proportion of monocytes in the d5 callus (**Figure 2D**). Monocytes can be phenotypically and functionally separated into classical (Ly6C^Hi^) and non-classical (Ly6C^Lo^) ( (34) and **Figure 2A**). The proportion of Ly6C^Lo^ non-classical monocytes was comparable in BM, unfractured bone, and d5 callus, and significantly reduced in d21 callus (**Figure 2F**). On the other hand, there was marked enrichment of the classical monocytes in both unfractured bone and d5 callus relative to BM or d21 callus (**Figure 2G**).

Macrophages are typically produced from two sources: yolk sac or liver progenitors during embryonic development, or following differentiation from monocytes. The F4/80^+^ macrophages in the calli of d5 or d21 were present at proportions indistinguishable from those found in unfractured bone or BM (**Figures 2E, H**). When we examined the proportion of activated callus macrophages, using MHC Class II and costimulatory molecules as a measure of activation, we found that the proportion of MHC Class II^+^ macrophages in the d5 and d21 callus was substantially below that found in BM, and similar to that found in bone (**Figure S9A**). However, the proportion of macrophages expressing the costimulatory molecules CD80 (**Figure 2I**), GITRL (**Figure 2J**), CD40 (**Figure S9B**) and CD86 (**Figure S9C**), as well as the expression levels of MHC Class II (**Figure 2K**), were all higher in macrophages that populated the d5 callus as compared to those that populated unfractured bone and BM, indicating that macrophages become activated during the inflammatory phase of fracture healing. Expression of MHC Class II and all the costimulatory molecules were significantly reduced on d21 vs d5, but the proportion of CD80^+^ (**Figure 2I**) and CD40^+^ (**Figure S9B**) macrophages, as well as levels of MHC Class II (**Figure 2K**), remained higher on d21 relative to BM.

### Specific dendritic cell subpopulations are enriched and activated in the healing callus during the early inflammatory phase

**Figure 3:**
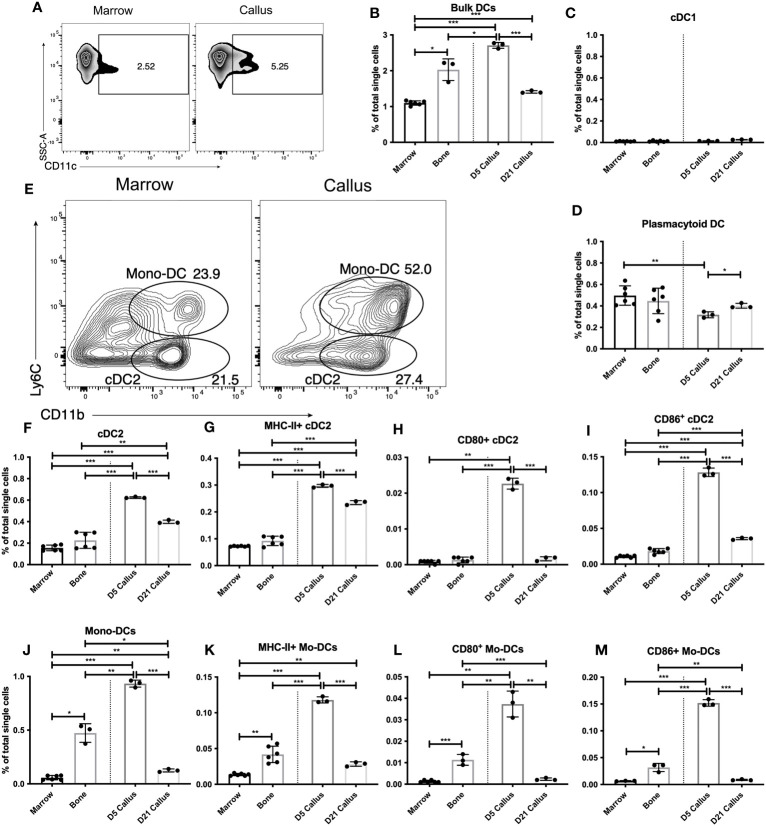
Distinct dendritic-cell subsets are enriched and activated in the early healing callus. Single cell populations liberated from marrow, bone or callus on day 5 (d5) or d21 post-fracture were analyzed by FC using the gating strategies outlined in **Table S8** and shown in **(A, E)** Quantification of **(B)** bulk DCs, **(C)** cDC1, **(D)** pDC, **(F)** cDC2, and **(J)** Mono-DCs as a proportion of total single cells in each population. Activation of DC populations was assessed by the proportion of singlets expressing MHC Class II **(G, K)** or the costimulatory molecules CD80 **(H, L)** or CD86 **(I, M)**. N = 3 for callus tissues (5 calli were pooled in each replicate); N = 3 – 6 for bone and BM. The bar graphs present average ± SEM. (*) *P* < 0.05, (**) *P* < 0.01, and (***) *P* < 0.001 using one-way ANOVA followed by Tukey’s *post hoc* test.

We expanded our analysis to dendritic cells (DC), which are the most closely related populations to the monocyte/macrophage axis. DC act as a crucial linkage between the innate and adaptive immune response (35). DC sense tissue damage, become activated, and either present antigen to T lymphocytes (which subsequently aid in B lymphocyte responses) or mediate DC-specific effector functions (35). As DC are a heterogenous cell population, we analyzed bulk DC as well as subpopulations (defined in **Table S8**). We found that bulk DC constitute ~1% of the BM single cell population (**Figures 3A, B**) a proportion consistent with DC in secondary lymphoid organs such as spleen or lymph node (36). Interestingly, bulk (i.e. CD11c^+^) DC were enriched in bone vs. BM, and further enriched in the d5 callus (~2.5-fold higher in the d5 callus than in the BM) (**Figures 3A, B**). However, by d21 the proportion of DC in the callus fell significantly relative to d5 (**Figure 3B**).

We then analyzed conventional and effector DC subpopulations. Within the conventional DC (cDC) population, cDC1 are primarily resident within secondary lymphoid organs and are important in initiating CD8^+^ T cell responses (37). As expected, we found few cDC1 in BM, bone or the callus (**Figure 3C**). Plasmacytoid DC (pDC) are effector DC that are primed to rapidly produce Type I interferons (38), which can play an important role in bone biology (39). We observed no enrichment of pDC in the fracture callus vs BM or bone (**Figure 3D**). An analysis of the proportion of pDC that express MHC Class II revealed no discernable increase in the d5 or d21 callus over unfractured bone, but showed a mild, yet statistically significant, increase in d21 callus over BM and d5 callus (**Figure S10A**). We also observed a mild yet significant increase in the levels of MHC Class II on the surface of pDC in the calli of d5 and d21 relative to either BM or unfractured bone (**Figure S10B**). In contrast, the proportion of PDCs expressing the costimulatory molecules (**Figure S10C** for CD40; data not shown for other costimulatory molecules) was comparable in unfractured bone, d5 callus, and d21 callus, and was substantially higher in the 3 tissues than in BM. Therefore, given the inconsistent expression patterns of the markers of DC activation, there is no evidence of enrichment or activation of pDC during fracture healing vs normal homeostatic conditions.

To explore the DC subpopulations that are responsible for the increased bulk DC infiltration we observed on d5, we examined the cDC2 (**Figures 3E, F**) and the monocyte-derived DC (Mo-DC) (**Figures 3E, J**) subpopulations. cDC2 migrate between peripheral tissues and secondary lymphoid organs and are important players in the initiation of CD4^+^ T cell responses (40, 41). There was a marked enrichment of cDC2 in the d5 callus (**Figure 3F**). The proportion of cDC2 was reduced in the d21 callus but remained substantially higher than that in the BM or bone (**Figure 3F**). On d5 and d21, a higher proportion of cDC2 in the callus than in the BM or bone expressed MHC Class II (**Figure 3G**) at elevated levels (**Figure S11A**). The proportion of CD80^+^ (**Figure 3H**), CD86^+^ (**Figure 3I**), CD40^+^ (**Figure S11B**), and GITRL^+^ (**Figure S11C**) cDC2 was substantially higher in the callus of d5 than in BM or unfractured bone. Meanwhile, the proportion of cDC2 that expressed most of these costimulatory molecules decreased on d21 to the background levels detected in BM and bone (**Figures 3H, I**, **S11B, C**), indicating recruitment and activation of cDC2 during the initial inflammatory phase of healing, which then reduced to background levels during the later phases.

Mo-DC (**Figures 3E, J**) are effector DC that differentiate from inflammatory monocytes. They produce TNFα and reactive nitrogen species (42) and can also alter functional differentiation of T lymphocytes locally (43, 44). The proportion of Mo-DC in the d5 callus was markedly higher than that in bone, which in turn was substantially higher than that in the BM (**Figure 3J**). Indeed, the increased proportion of cDC2 and Mo-DC cumulatively accounted for almost all the increase in the bulk DC population that we detected in d5 callus (**Figure 3B**). There was also a marked increase in the proportion of MHC Class II^+^ (**Figure 3K**), CD80^+^ (**Figure 3L**), CD86^+^ (**Figure 3M**), CD40^+^ (**Figure S12A**), and GITRL^+^ (**Figure S12B**) Mo-DC, as well as the levels of MHC Class II (**Figure S12C**), in the d5, but not in the d21, callus relative to bone or BM. Interestingly, the proportion of Mo-DC expressing MHC Class II and all the aforementioned co-stimulatory molecules was substantially higher in unfractured bone than in the BM (**Figures 3K-M**, **S12A, B**). Therefore, similar to cDC2, Mo-DC are recruited and activated during the early phases of healing. It is noteworthy that the proportion of MHC Class II^+^ Mo-DC in the d5 callus was ~40% of that in the cDC2 population (**Figures 3G, K**), but the number of Mo-DC was larger than that of cDC2 population (**Figures 3F, J**). Taken together, our results demonstrate that innate immune cells, primarily monocytes, macrophages, and DC, are activated during the inflammatory phase of fracture healing and return to close to near-homeostatic numbers and activation status during the later phases. To corroborate these results, we used RT-qPCR to measure the expression level of (C-X-C motif) ligand 16 (Cxcl16), a chemokine secreted at high levels by activated macrophages and DC. Cxcl16 expression on days 10 and 21 was 3-4-fold less than its expression on days 3 and 5 (**Figure S12D**), confirming that recruitment and activation of innate immune cells occur mainly during the early repair phases.

### The lymphoid response in the fracture callus, unfractured bone, and bone marrow

**Figure 4:**
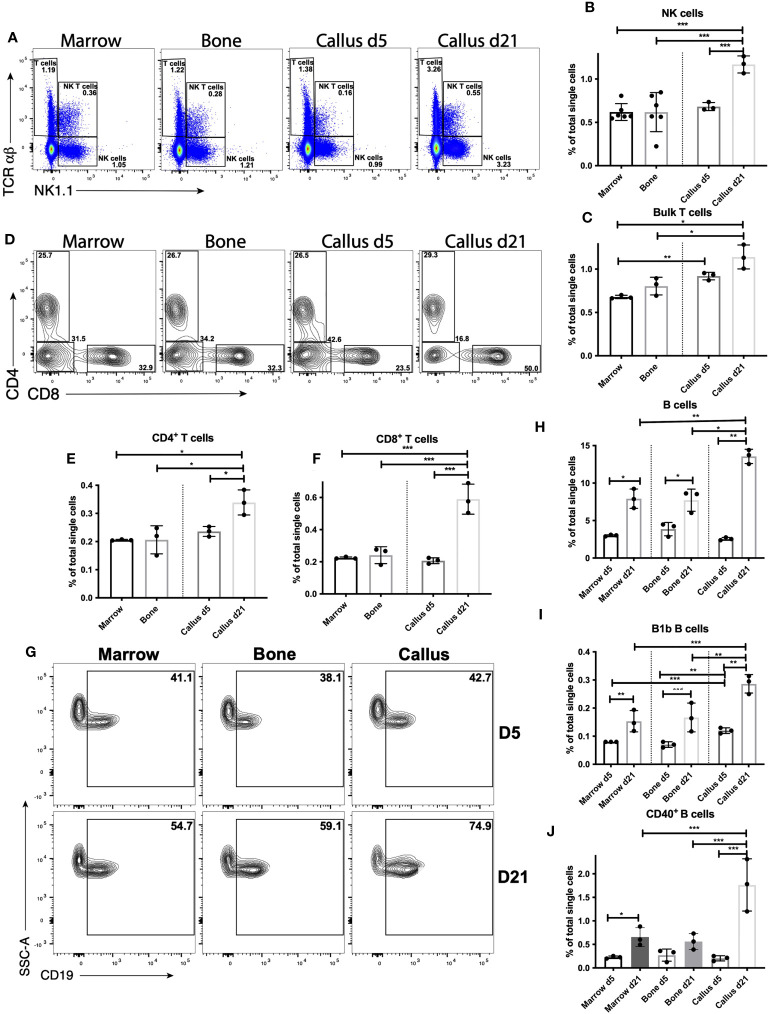
Lymphocyte response peaks during the late repair phase of fracture healing. Single cell populations liberated from marrow, bone or callus on day 5 (d5) or d21 post-fracture were analyzed by FC using the gating strategies outlined in **Table S8** and shown in **(A)** (NK cells, NK T cells and T cells), **(D)** (CD4^+^ and CD8^+^ T cells) and **(G)** (B cells). Quantification of **(B)** NK cells, **(C)** bulk T cells, **(E)** CD4^+^ T cells, **(F)** CD8^+^ T cells, **(H)** B cells, and **(I)** B1b B cells as a proportion of total single cells in each population. Activation of B cells was assessed by measuring the proportion of singlets expressing the costimulatory molecule CD40 **(J)**. N = 3 for callus tissues (5 calli were pooled in each replicate); N = 3 – 6 for bone and BM. The bar graphs present average ± SEM. (*) *P* < 0.05, (**) *P* < 0.01, and (***) *P* < 0.001 using one-way ANOVA followed by Tukey’s *post hoc* test.

Because myeloid and DC responses shape the subsequent lymphoid response, in which Natural Killer (NK) cells (45–48), T cells (14–16, 49) and B cells (16, 17) are known to impact fracture healing, we investigated the proportion of lymphoid cells in the fracture callus (phenotypes are outlined in **Table S8** and shown in **Figures 4A, D, G**). We started with the analysis of d5 callus and found no significance difference in the proportion of NK cells there vs. in either bone or BM (**Figures 4A, B**), which indicates re-establishment of the normal proportion of NK cells found in bone/BM in the healing hematoma. The proportion of NK T cells, a heterogenous population of cells that share a number of properties of both T cells and NK cells, in the callus was comparable to that in bone and significantly less than the proportion in BM (**Figure S13A**). On the other hand, the proportion of bulk T cells was slightly, but significantly, higher in the d5 callus than in the BM (**Figures 4A, C**). The T cell population is generally composed of CD4^+^ and CD8^+^ T cells, but bone marrow is enriched in a CD4^-^ CD8^-^ T cell subpopulation (50, 51). Analysis of these T-cell subpopulations indicated comparable proportions of CD4^+^ and CD8^+^ T cells in the d5 callus vs bone or BM (**Figures 4D-F**). The only T-cell subpopulation whose proportion was higher in the callus than in the BM or bone was the CD4^-^ CD8^-^ T cells (**Figure S13B**), which might account for the small increase in the bulk T cells observed in the d5 callus relative to the BM (**Figure 4C**). Analysis of B cells indicated comparable proportions of bulk B cells in d5 callus, bone, and BM (**Figures 4G, H**). We also analyzed the B-cell subpopulation B1b B cells, a subset of innate lymphoid cells that displays a wider specificity and responds earlier after insult than conventional B cells. We observed a small, yet significant, increase in the proportion of this subpopulation in the d5 callus vs. bone and bone marrow (**Figure 4I**). Collectively, these results indicate that the normal proportions of lymphocytes that exist in unfractured bone/BM are re-established in the healing hematoma of d5.

In contrast to the myeloid and DC response, which was increased on d5 and reduced on d21, the proportions of NK (**Figures 4A, B**) and bulk T cells (**Figures 4A, C**) increased on d21 relative to d5, and the proportions of both populations were substantially higher in d21 callus than in BM or bone (**Figures 4A-C**). Unlike on d5, the increase observed in the proportion of bulk T cells on d21 could be accounted for by an increase in CD4^+^ (**Figure 4E**) and CD8^+^ (**Figure 4F**) T cells, whereas the proportion of CD4^-^ CD8^-^ T cells was reduced on d21 vs d5 (**Figure S13B**). Interestingly, the proportion of B cells was dramatically (~450%) increased in the d21 callus relative to the d5 callus, but also increased in the BM (~ 260%) and bone (~200%) harvested from d21 mice as compared to those harvested from d5 mice (**Figures 4G, H**). Notably, the proportion of B cells in d21 callus remains significantly higher than their proportions in bone or BM (**Figures 4G, H**). In addition, B1b B cells expanded with similar kinetics in all tissues analyzed (**Figure 4I**). Therefore, these data suggest that there is a systemic conventional B cell response triggered by bone fracture. We also examined activation of B cells, measured by upregulation of the costimulatory molecules CD40 and CD86. In doing so, we detected upregulation of CD40 expression in (~13%) of B cells in the d21 callus, which was ~150-fold higher than the proportion of CD40^+^ B cells in BM (~0.08%) or bone (~0.07%) (**Figure 4J**). A much smaller proportion of B cells upregulated CD86 (**Figure S13C**) and other costimulatory molecules (not shown). In summary, our data support a strong lymphoid response in the NK and T cell compartment in the d21 callus, with a much smaller response in any lymphoid compartment during the early inflammatory phase. Consistently, RT-qPCR analysis showed 4- to 9-fold increase in the expression of C-C motif chemokine ligand 5 (Ccl5) and interferon gamma (Ifng), both of which are highy produced by activated T cells, on d21 relative to days 3, 5, and 10 (**Figures S13D, E**). Notably, the expression of tumor necrosis factor (Tnf) also increased 2 to 6 fold on d21 relative to days 3, 5, and 10 (**Figure S13F**). Importantly, the lymphoid response observed on d21 cannot be attributed to re-establishment of BM elements at this timepoint, as the callus contained cells that were highly enriched compared to the BM. The B cell response on d21 becomes systemic but is greatly enhanced in the callus at this timepoint.

### Defective healing in DIO mice is accompanied by a dysregulated pattern of immune response

**Figure 5:**
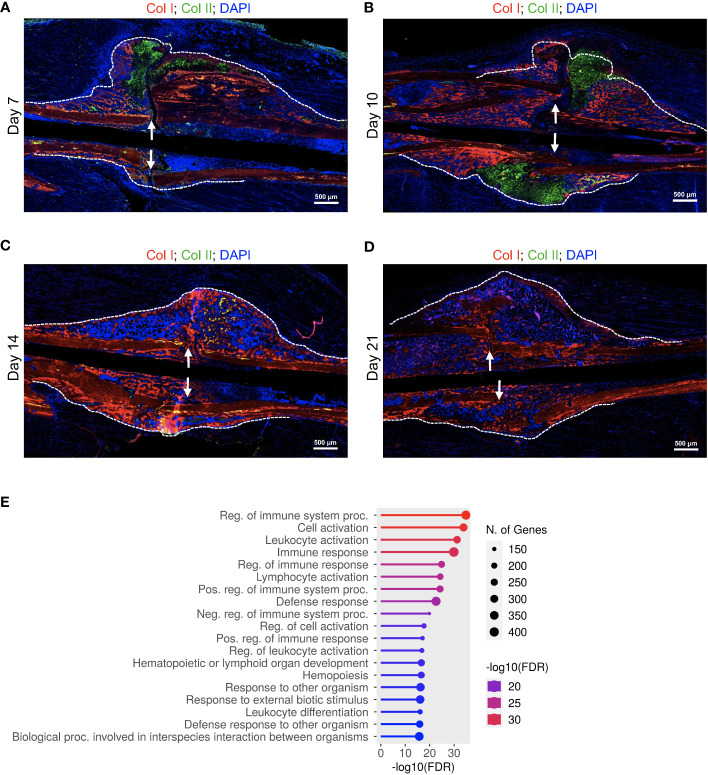
Delayed fracture healing in DIO mice is accompanied by a defective immune response. **(A-D)** IF co-staining of Col I (red) and Col II (green) in callus tissues harvested at the indicated timepoints. Col II is a chondrocyte marker, while the red staining in the callus marks the newly formed bone. DAPI stains nuclei (blue). The scale bar = 500 μm. All images are representative of 5 callus tissues (see also **Figure S14** and (3)). **(E)** GO analysis of genes that showed significantly different time-course expression between lean and DIO mice (**Table S10**). RNA-seq was performed on 3 mice at each timepoint.

We reported previously that DIO mice exhibit defective fracture healing that is typified by accelerated/pre-mature resorption of the soft callus (3). Accelerated soft callus resorption can be observed by comparing representative images of lean callus (**Figures 1A-C**; **S1A-C**) and DIO callus (**Figures 5A-C**; **S14A-C**). DIO mice also show reduced bone formation (compare **Figures 1D**; **S1D** to **Figures 5D**; **S14D**) and aberrant structure of collagen fibers (3). To gain a more comprehensive understanding of fracture healing in DIO mice, we performed RNA-seq using the healing calli of DIO mice at the same timepoints we used with the lean mice. We analyzed the overall pattern of gene expression over the time-course of healing in both lean and DIO groups by applying a generalized linear model fitted with a negative binomial distribution, followed by linear regression to model gene expression over the healing time-course (**Table S9**). We identified 3909 genes that exhibited significantly different expression patterns over the course of healing in lean vs. DIO mice (**Table S9**). Gene ontology (GO) analysis demonstrated that these genes are primarily enriched in pathways involved in immune response and leukocyte activation (**Figure 5E**; **Table S10**). These data indicate that the pattern of immune response in DIO mice during the healing course deviates from the normal pattern in lean mice. Comparison of individual days indicated that DIO mice exhibited defective immune responses and reduced activation of several immune system components between days 3 and 7 post-fracture (**Figures S15-17**; **Tables S11**-**16**). Importantly, the most significant changes in the expression of genes involved in immune response and leucocyte activation between lean and DIO mice were observed on d5 (**Figure S16**, **Tables S13**, **14**). On the other hand, DIO mice showed accelerated chondrocyte differentiation, cartilage development, and upregulated proteoglycan biosynthesis on days 5 and 7 (**Figures S16, 17**; **Tables S13**-**16**).

Consistent with our published data (3) and histological analysis (**Figures 1A-C**, **5A-C**, **S1A-C, S14A-C**), DIO mice exhibited accelerated/premature resorption of the soft callus as evidenced by significantly reduced expression of genes involved in chondrocyte differentiation, cartilage development, and production of cartilage extracellular matrix relative to lean mice on day 10 (**Figure S18**; **Tables S17**, **18**). Analysis of the chondrocyte markers Aggrecan (Acan) and collagen, Type II, alpha 1 (Col2a1) demonstrated that the expression of both genes peaked earlier in the callus of DIO mice (**Figure S18C**), confirming accelerated formation of the soft callus in DIO mice. On d10, when the expression of both Acan and Col2a1 peaked in the lean callus, the mRNAs of both genes declined sharply in the DIO callus, further confirming our published data (3) and histological analysis (**Figures 1A-C**, **5A-C**; **S1A-C, S14A-C**) showing comparatively earlier resorption of the soft callus in DIO mice.

Importantly, the early resorption of the soft callus in DIO mice was accompanied by reduced bone morphogenesis, which occurred as early as d10 (**Figure S18B**; **Table S18**). The calli of DIO mice exhibited abnormally upregulated differentiation of fat cells on d10 (**Figure S18B**; **Table S19**), consistent with published data showing increased adipogenic commitment of mesenchymal stromal cells in DIO mice (23). Intriguingly, the calli of DIO mice showed clear and extensive inhibition of translation and ribosome biosynthesis on d14 (**Figure S19**; **Tables S20**, **21**). While lean mice showed substantial upregulation in the expression of genes involved in immune response between days 14 and 21 (**Figures 1E, F**, **S7A-C**; **Tables S6**, **S7**), DIO mice showed significant reduction in the immune response on d21 relative to lean mice (**Figure S20**. **Tables S22**, **23**). Furthermore, while genes involved in ossification and mineralization peaked on d14 in the callus of the lean mice (**Figures S7A, B**; **Table S7**), these genes showed delayed expression in the callus of the DIO mice (**Figure S20**; **Tables S22**, **23**). Taken together, the RNA-seq data corroborated the results of the IF staining and histological analyses and revealed additional defects in the healing callus of DIO mice, including defective immune responses during the early inflammatory phase as well as during the second infiltration wave of immune cells between days 14 and 21.

### Cell-type-specific changes in the myeloid response in the fracture callus of DIO mice

**Figure 6:**
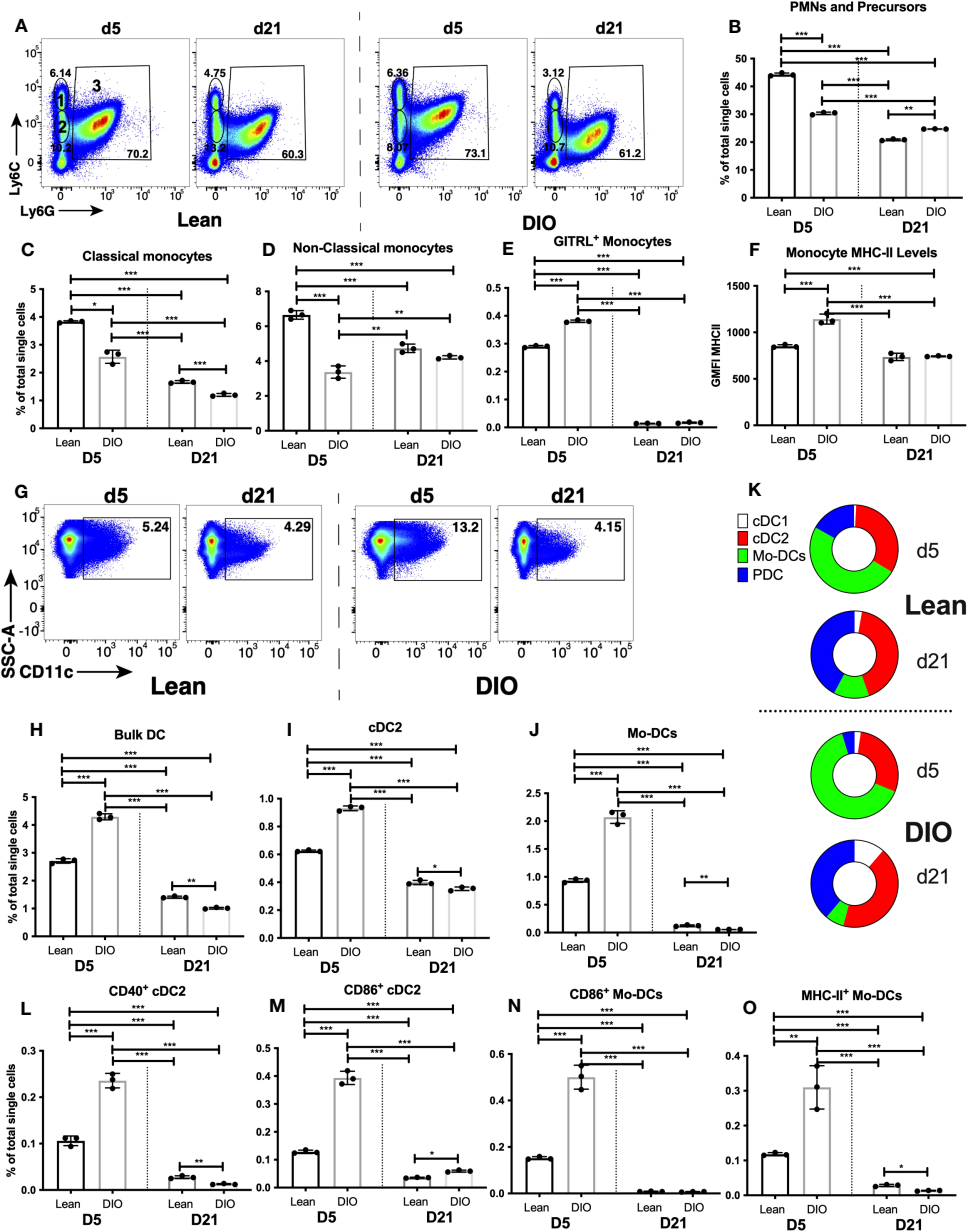
DIO mice exhibit dysregulated innate immune responses during the early inflammatory phase of fracture healing. Single cell populations liberated from marrow, bone or callus on day 5 (D5) or D21 post-fracture were analyzed by FC using the gating strategies outlined in **Table S8** and shown in **Figure 2A** and **(A)** (PMNs, classical and non-classical monocytes) and **Figure 3A** and **(G)** (DC). Quantification of **(B)** PMNs and PMN precursors, **(C)** classical monocytes, **(D)** non-classical monocytes, **(H)** Bulk DC, **(I)** cDC2, and **(J)** Mo-DCs as a proportion of total single cells in each population. **(K)** Quantification of DC subpopulations (cDC1, cDC2, pDC and Mo-DC) as a proportion of the total bulk DC populations. Activation of monocytes was assessed by measuring the proportion of singlets expressing the costimulatory molecule GITRL **(E)** or cell surface levels of MHC Class II **(F)**. Activation of DCs was assessed by measuring the proportion of singlets expressing the costimulatory molecules CD40 **(L)**, CD86 **(M, N)** or MHC Class II **(O)**. N = 3 for all tissues. For callus analysis, 5 calli were pooled in each replicate. The bar graphs present average ± SEM. (*) *P* < 0.05, (**) *P* < 0.01, and (***) *P* < 0.001 using one-way ANOVA followed by Tukey’s *post hoc* test.

To get more cell-specific details on the dysregulated pattern of immune response in DIO mice that we identified using RNA-seq data, we directly compared the proportions of myeloid cell types (examined in **Figures 2**, **3**) in the calli of lean and DIO mice on days 5 and 21. The largest population of immune cells in the callus, PMNs and myeloid cell precursors, was reduced ~30% in the d5 callus of DIO mice relative to lean mice (**Figures 6A, B**), a reduction that ablated the small increase observed in this population in the d5 callus of lean animals relative to bone and BM (**Figure 2B**). On d21, the proportion of PMNs and myeloid cell precursors was statistically higher in DIO mice, although the proportions in both lean and DIO remained lower than those found on d5 (**Figures 6A, B**). On d5, the proportion of total monocytes was also lower in DIO mice (**Figure S21A**), and this was accounted for by a drop in both classical (**Figure 6C**) and non-classical (**Figure 6D**) monocyte populations, with only minor differences in the monocyte proportions between lean and DIO calli on d21 (**Figures 6C, D**, **S21A**). Conversely, there was an increase in the proportion of activated monocytes in the d5 callus of DIO mice relative to lean mice as indicated by an increase in the proportion of monocytes expressing the costimulatory molecule GITRL (**Figure 6E**) and in the level of surface expression of MHC-II (**Figure 6F**). However, the number of monocytes expressing the co-stimulatory molecule CD86 was decreased in the DIO callus relative to the lean callus at this timepoint (**Figure S21B**), while the proportion of monocytes expressing MHC-II was comparable in the lean and DIO calli (**Figure S21C**), a finding that complicates any conclusions regarding the activation status of monocytes in DIO relative to lean mice.

The F4/80^+^ macrophage population was not altered in DIO mice (on either d5 or d21) relative to lean mice (**Figure S21D**). The proportion of macrophages that became activated on d5 was greater in DIO mice than in lean mice, as measured by an increase in the cell surface levels of MHC-II (**Figure S21E**), and an increase in the proportion of MHC-II^+^ (**Figure S21F**), CD40^+^ (**Figure S21G**), CD80^+^ (**Figure S21H**), CD86^+^ (**Figure S21I**) and GITRL^+^ (**Figure S21J**) macrophages. Any activation of these cells had returned to background levels by d21 in both lean and DIO mice (**Figures 2I-K**, **S21E-J**).

DC were among the most highly modulated cell populations in the callus of lean mice on d5 (**Figure 3B**), and the proportion of bulk DC in the DIO callus at this timepoint was increased ~58% over that in lean mice, returning to baseline by d21 (**Figures 6G, H**). Of the DC subpopulations, the proportions of PDC (**Figure S22A**) and cDC1 (**Figure S22B**) in the DIO callus were comparable to those in the lean callus on d5 and d21. Interestingly, on d5, there was a ~60% increase in the proportion of cDC2 (**Figure 6I**) and a ~120% increase in the proportion of Mo-DC (**Figure 6J**) in DIO callus relative to lean callus. This created a stoichiometry of DC subpopulations (i.e., the % of each subpopulation relative to total DC not to total single cells) that was substantially different between lean and DIO calli on d5, with the most noticeable difference being the expansion of the Mo-DC subpopulation in the DIO callus to constitute ~64% of the total DC (**Figure 6K**). The increase in the proportions of cDC2 and Mo-DC in the DIO callus as compared to lean callus on d5 (**Figures 6I, J**) was accompanied by a greater activated proportion of each of the two subpopulations, as measured by an increase in the proportion of cells expressing CD40 (**Figure 6L**), CD86 (**Figures 6M, N**) and MHC-II (**Figure 6O**) (data not shown for other markers). The % of MHC-II^+^ and CD40^+^ PDC and the expression level of MHC-II on PDC were comparable in the lean and DIO calli (**Figures S22C-E**). By d21, the increase in DC response observed on d5 in DIO callus had normalized, and the proportions of almost all DC and activated DC subsets in the DIO calli were either similar to or lower than their proportions in the lean calli (**Figures 6H-O**; **S22A-E**). Notably, the stoichiometry of the DC subpopulations was substantially different between d5 and d21 in both lean and DIO mice: on d5, Mo-DC was the major DC subpopulation in both lean and DIO calli, while on d21, Mo-DC shrank in both lean and DIO calli to become a minor subpopulation, resulting in an increase in the % of all other subpopulations, especially PDC which, together with cDC2, were the major DC subpopulations on d21 (**Figure 6K**). In summary, during the inflammatory phase, while there is a reduction in PMNs and monocytes in the DIO callus relative to lean callus, there is an increase in the proportions of activated macrophages as well as an increase in the DC response, primarily cDC2 and Mo-DC. Consistent with this, RT-qPCR analysis detected a significant increase in the expression of Cxcl16 in the DIO callus relative to the lean callus on d5, but not at any later timepoint (**Figure S22F**).

### Reduction of the lymphoid response in the fracture callus of DIO mice

**Figure 7:**
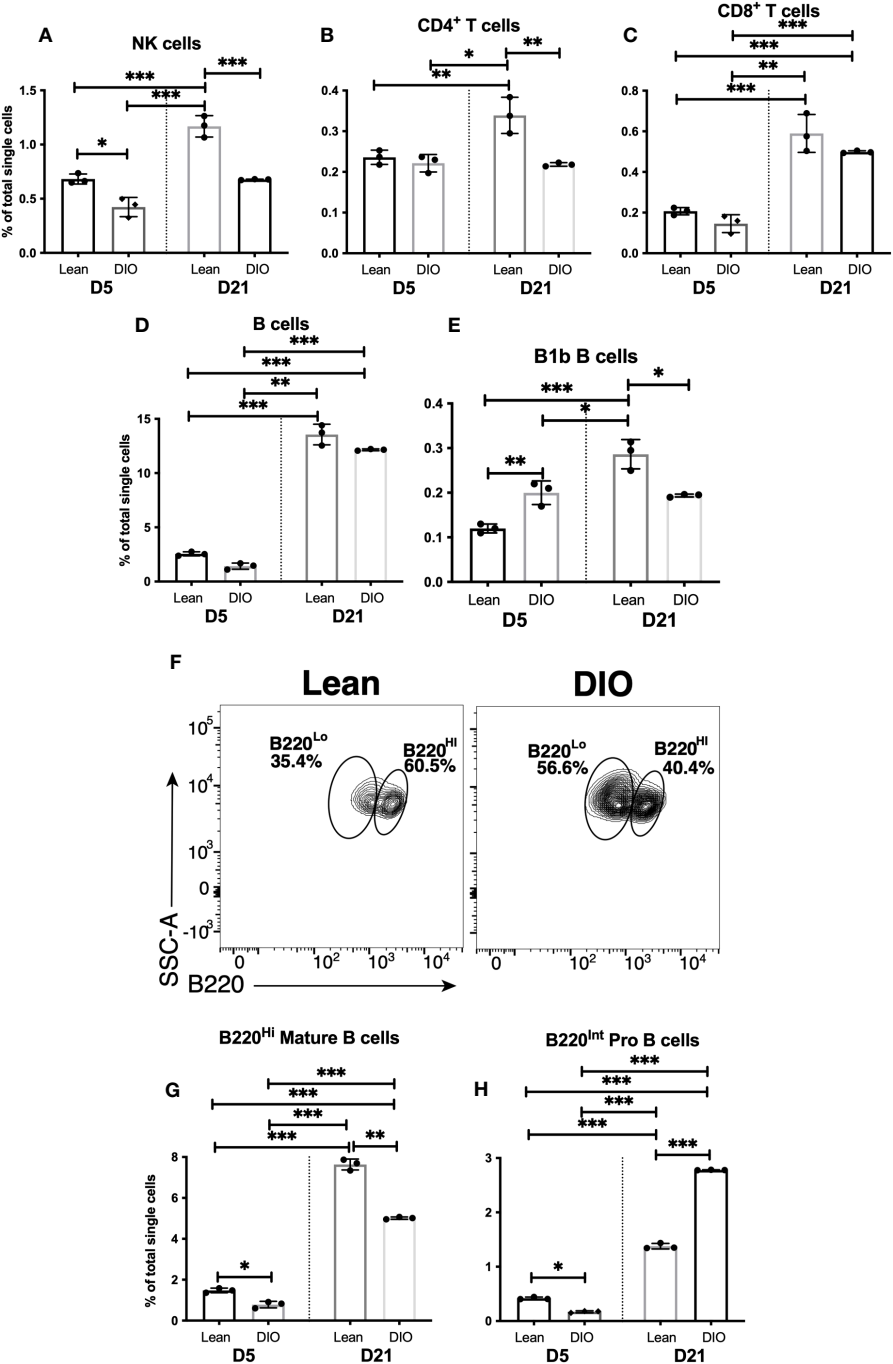
The adaptive immune response is blunted in the callus of DIO mice. Single cell populations liberated from fracture callus on day 5 (D5) or D21 post-fracture were analyzed by FC using the gating strategies outlined in **Table S8** and **Figures 4 A, D, G** Quantification of **(A)** NK cells, **(B)** CD4^+^ T cells, **(C)** CD8^+^ T cells, **(D)** B cells, and **(E)** B1b B cells as a proportion of total single cells. **(F)** Staining with B220 to reveal subpopulations of mature (B220^Hi^) and immature (B220^Lo^) B cells. Quantification of **(G)** mature and **(H)** immature B cells as a proportion of total single cells. N = 3 for all tissues. For callus analysis, 5 calli were pooled in each replicate. The bar graphs present average ± SEM. (*) *P* < 0.05, (**) *P* < 0.01, and (***) *P* < 0.001 using one-way ANOVA followed by Tukey’s *post hoc* test.

As we observed substantial changes in innate myeloid cell populations in the callus of DIO mice, we hypothesized that there would be likely changes in the lymphoid responses that are primed and shaped by the early myeloid cell responses. Therefore, we examined the proportion of NK cells, NK T cells, T cells and B cells in the DIO vs. Lean callus on days 5 and 21. The proportions of almost all the analyzed lymphoid cell populations on d5 in the DIO mice were similar to those in the lean mice, except for the innate B1b B cells whose proportion increased in the DIO callus over the lean callus at this timepoint (**Figures 7A-E**, **S23A, B**). On the other hand, many differences were observed between lean and DIO calli in the lymphoid populations on d21. The proportion of NK cells was significantly lower in DIO mice than in lean mice, and, unlike in the lean mice, the proportion of this population did not increase on d21 relative to d5 in the DIO mice (**Figure 7A**). There was no significant difference in the NK T cell population in the DIO callus vs lean callus (**Figure S23A**). In the T-cell compartment, relative to the lean mice, DIO mice showed a lower proportion of CD4^+^ (**Figure 7B**), but similar proportions of CD8^+^ T cells (**Figure 7C**) and CD4^-^CD8^-^ T cells (**Figure S23B**). No discernable difference was observed between lean and DIO calli in the proportion of the bulk B cells (**Figure 7D**). However, we did observe an alteration in the constituents of the B cell population. The proportion of the innate B1b B cells were reduced in the DIO callus as compared to the lean callus at this timepoint (**Figure 7E**). We also observed that, in the lean callus, the majority of B cells expressed high levels of CD45R (B220), indicative of a mature status (**Figures 7F, G**). In contrast, in the DIO callus, the proportion of mature cells was reduced and replaced by immature B cells expressing intermediate levels of CD45R, which likely represent Pro B cells that are not fully functional (**Figures 7F, H**). Therefore, in the DIO callus there is an enhanced early d5 B1b B cell response, but deficits in the NK, CD4+ T, B1b B cell, and mature B cell responses on d21, indicating that the adaptive immune response observed on d21 in lean mice (**Figure 4**) is defective in DIO mice. RT-qPCR results were in total agreement with this conclusion and showed significantly reduced expression of Ccl5, Ifng, and TNF on d21 in the callus of DIO mice as compared to the callus of lean mice (**Figures S23C-E**).

## Discussion

Non-unions and impaired fracture healing afflict millions in the US every year, which necessitates surgical interventions for hundreds of thousands of these patients and thereby imposes a huge burden on the health care system (2). The rate of non-union development during fracture healing increases significantly with certain comorbidities, including metabolic diseases such as type 2 diabetes (T2D) (52); accordingly, the growing incidence of obesity/T2D makes impaired fracture healing an increasing concern (53, 54). Importantly, obese and T2D patients exhibit systemic, as well as tissue-specific, alterations in the response and functions of immune cells (55). Based on these facts, along with published reports on the central roles of different immune cells in the fracture healing process (2), immunomodulatory agents have emerged as an attractive therapeutic approach to address impaired healing (2). However, efforts aimed at defining novel targets for immuno-therapeutic interventions have been hindered by limited information and fragmented knowledge regarding the repertoire of immune cells that populate the callus during different phases of healing. Here, we comprehensively define the composition of immune cells that populate the normal callus at 2 different timepoints, representing 2 phases during which extensive immune-cell infiltration into the healing callus was observed, and we show how these immune cell populations differ from those found in bone and BM. Furthermore, we provide a comprehensive comparison between the stoichiometry of immune cells in the callus of lean vs DIO mice at the same 2 timepoints, which will aid in the identification of new therapeutic targets for immunomodulatory agents.

The immune system is generally thought to have evolved to protect against pathogen challenge, but the positioning of immune cells in every tissue, along with the ability to recruit immune cells rapidly from the circulation, allows it to play a pivotal role in maintenance of tissue health. Bone is a unique tissue as it constitutes an osteoimmune system: bone acts as a locomotor organ and a reservoir of minerals, and, simultaneously, the host organ for the BM where hematopoietic stem cells reside (56). The field of osteoimmunology continues to uncover new dimensions of the reciprocal interaction between bone and the immune system (2, 57). Maintenance of normal bone homeostasis and regeneration requires proper function of bone cells that include osteoblasts, osteocytes, and osteoclasts. The actions of these cells are regulated by immune cells and can be negatively impacted by aberrant inflammatory or immune-cell milieu that is associated with specific pathological conditions (57). From the data we show here, it is clear that there are numerous complex steps through which immune components aid in bone regeneration. Our initial RNA-seq time course revealed that there are two major waves of immune and inflammatory activity in the healing callus, with a previously appreciated peak of activity on d5, and an additional, previously unidentified peak on d21 (**Figures 1E; F**, **S2-7**). The RNA-seq analysis gave a high-level view of immune activation, illustrating enhanced expression of genes associated with immune response, immune system process, defense response, and response to other organisms. Our subsequent FC analysis complemented the RNA-seq results and revealed that, perhaps as one might expect, the processes involved on d5 and d21 are modulated by activation and expansion/recruitment of different immune cell types (**Figures 2**-**4**; **S8-S13**). Taken together, our data reveal the dynamic nature of the repertoires of immune cells that populate the callus during different phases of healing, consistent with the complex cellular makeup of the callus and the underlying biological processes that constantly change from one healing phase to another. Our data additionally identified immune-cell populations that are specifically enriched/activated in the callus as compared to unfractured bone/BM (**Figures 2**-**4**; **S8-S13**).

A typical immune response to a pathogen is thought to involve initial recruitment of PMNs, followed by monocyte recruitment and the concurrent migration of tissue resident DC away from the site of insult to secondary lymphoid organs, where activation of T and B lymphocytes occurs. Some aspects of the lymphocyte response can occur early, such as activation of B1b B cells and NK cells which, although lymphocytes, can be activated much earlier and by a wider range of stimuli than conventional T and B cells (58, 59). We did observe an early enrichment of B1b B cells (**Figure 4I**), but not NK cells (**Figure 4B**), in the healing hematoma of d5 over their normal proportions in BM or unfractured bone, suggesting a specific role of B1b cells in an early innate-like response. Except for B1b B cells, this early expansion of immune-cell populations beyond their homeostatic levels in BM/Bone was restricted primarily to cells of the myeloid lineage, which are not antigen-specific and are specialized to recognize, and be activated by, the release of Danger Associated Molecule Patterns (DAMPs) from damaged cells like those present post-fracture (60, 61)). Cells identified as PMNs and myeloid cell precursors were the major immune cell population in the d5 callus and were enriched over their normal levels in bone or BM (**Figure 2B**), consistent with published data reporting that PMNs, as well as PMN release of neutrophil extracellular traps, enhance fracture healing via a mechanism that is still being elucidated (4–6, 27, 62). Neutrophils play roles in fibrin thrombus formation during the early inflammatory phase (28), contribute to removal of tissue debris during later stages of inflammation (4, 63), and secret cytokines that recruit monocytes to the fracture callus (1, 5, 6). Our data show that by d21 PMNs were present at much lower levels than those found in bone or BM (**Figure 2B**), likely because the presence of reactive oxygen species at this stage of healing is less required than in the early healing phases. Notably, this reduced abundance of PMNs underscores the unique microenvironment of the BM that exists in the callus of d21 compared to that of the BM in unfractured bone.

Several publications reported important roles for monocytes or macrophages in the fracture healing (2, 8, 10–13). Monocytes can differentiate into either M1 or M2 macrophages (64). M1 inflammatory macrophage secret cytokines that recruit more monocytes and contribute to removal of necrotic cells and fibrin thrombus (10). On the other hand, M2 macrophages play anti-inflammatory roles during the later stages of the inflammatory response and secret proteins that promote osteogenesis and angiogenesis (2, 12, 13). We observed a comparable abundance of monocytes and macrophages in callus, bone, and BM (**Figures 2H**; **S8A**), but elevated level of macrophage activation in the callus vs other tissues, (**Figures 2I-K**; **S9B, C**) consistent with a role for macrophages in fracture repair. A recent study examining macrophages, defined as CD45^+^, CD11b^+^, CD11c^Int/-^, and CD68^+^, has described a large infiltration of macrophages to the hematoma at d3 post fracture, just prior to the d5 time point we have examined (7). However, we believe our observation of a similar abundance of monocytes and macrophages in the callus, bone, and BM support a different interpretation of the cells present in the callus in light of the added complexity of our FC analysis. Specifically, we have examined DC and DC subpopulations, including mo-DC, cDC1 and cDC2, in the healing callus (see below), an approach that has yet to be employed by other researchers in the field.

The myeloid cell compartment is derived from one of the most plastic developmental programs in the body. A common myeloid progenitor (CMP) can produce all the myeloid lineages and can also produce cells that combine the characteristics of multiple downstream cell types. One such cell type is the granulocytic myeloid derived suppressor cell, which expresses a set of cell surface proteins that combine the markers of typical monocytes and neutrophils (65), i.e., these cells could readily be mistaken for either neutrophils or monocytes, depending on the depth of the analysis conducted. Another cell type of this kind is Mo-DC, which differentiate from monocyte precursors into CD11c^+^ DC and can activate T cell populations locally. Unless examined in the context of molecules expressed specifically by DC, these cells could readily be identified as monocytes or macrophages; therefore, we believe that the increased monocyte/macrophage response reported previously is likely to have included a component of Mo-DC response. Indeed, the use of staining for CD68, a lysosomal protein macrosialin, in a recent study (7) does not distinguish macrophages and monocytes. In addition, the exclusion of CD11c^Hi^, but not CD11c^Int^, cells from the macrophage gate means that Mo-DCs are included in the macrophage population, so our data are likely consistent with those observed by Bucher et al. (7). As future technological advances enable further differentiation of discrete cell types, it is likely that the classifications of myeloid cells we are currently using will be surpassed in favor of others with greater specificity and functional differentiation. For now, however, we are confident to state that the Mo-DC response (**Figure 3J**), combined with a smaller magnitude response by cDC2 (**Figure 3F**), indicate a substantial recruitment or proliferation of these cells in the callus.

Mo-DC in the callus could play a role in activation of locally resident T cell populations, but Mo-DC are also known to produce large quantities of TNF and reactive nitrogen species, each of which could play an important role in the early inflammatory response after fracture. Multiple cell types do express MHC Class II in the healing callus (**Figures 2C, K**, **3G, K**), but the specialized phenotype of DC renders them substantially more effective at initiating either a memory or a primary T cell response (35). Therefore, these cells likely play a requisite role in initiation of the T cell response known to be required for efficient fracture healing (14–16, 49). Although the MHC-II levels on Mo-DC are upregulated (**Figure S12C**), these expression levels pale in comparison to the upregulation of MHC-II on the surface of cDC2 (**Figure S11A**), which represents the highest level of any cell analyzed in bone, BM or d5 callus (not shown). It is possible that these cells also activate T cell populations locally, although the most accepted role for DC is to internalize antigen at a site of insult, and then traffic to secondary lymphoid organs where naïve T cells populations are activated. Examining DC migration from an internal organ to secondary lymphoid organs is technically challenging, so our study is currently unable to conclude that this occurs, or if it is an important factor in fracture healing. Notably, both the proportion and activation of both Mo-DC and cDC2 returned to near-background levels by d21 (**Figures 3F–M**), when initiation of the adaptive immune response is no longer required. The large difference in the stoichiometry of DC subpopulations between d5 and d21 (**Figure 6K**), especially the extensive shrinkage of Mo-DC on d21 vs d5, indicates that the type of DC response required during the initial inflammatory phase is substantially different than that required during the late phases of healing.

Our RNA-seq analysis did indicate expression of genes involved in T cell activation during the early wave of immune activity on d5 (e.g., lymphocyte protein tyrosine kinase [Lck] and CD3 antigen, epsilon polypeptide [Cd3e]) (**Figures S24A, B**). At this timepoint, the numbers and proportions of classical CD4^+^ and CD8^+^ T cell populations, NK cells, and B cells mirrored their normal abundance in bone and BM (**Figure 4**). During this phase of healing, T cells are reported to secret tumor necrosis factor (ligand) superfamily, member 11 (Tnfsf11, also known as RANKL) that promote osteoclast differentiation and activation, which in turn contribute to removal of the fibrin thrombus toward the end of the inflammatory phase (2, 66). B cells also paly roles during this phase as they suppress the proinflmatory signals (17) and secret tumor necrosis factor receptor superfamily, member 11b (Tnfrsf11b, also known as OPG) to regulate differentiation and activation of osteoclasts (18, 66). Our data show reproducible increase in the proportion of bulk T cells in d5 calls relative to BM (**Figure 4C**), which can be accounted for by expansion or recruitment of a poorly characterized CD4^-^ CD8^-^ population that has been described in BM (**Figure S13B**) (50, 51). As in the myeloid cell populations described above, this may reflect a population that is driven from precursors by local conditions. As CD4^+^ or CD8^+^ T cells of any individual specificity may number in the low hundreds per mouse (67), massive expansion of those cells is required to detect an increase in any cell population. Such expansion and migration during the early inflammatory phase, likely by the large number of DC, could account for the increased number of CD4^+^ or CD8^+^ T cells on d21 as compared to d5 (**Figures 4E, F**), the clear increase in expression of genes involved in T-cell activation (**Figures S24A, B**), and the increase in cytokines and chomkines expressed by activated T cells (**Figures S13D, E**). Alternatively, activation of local populations of cells may occur during the early inflammatory phase, with recruitment of cells primed in secondary lymphoid organs during the second phase. This could likely explain the two waves of infiltration and the proposed differential functions of T cells during the inflammatory as well as mineralization/remodelling phases (15, 16, 68)

B cells have been implicated in efficient fracture healing (16, 17). The proportion of innate B1b B cells is increased in the callus at d5 over their levels in BM or bone (**Figure 4I**). However, all other B cells follow a similar time course to other lymphocyte populations: B cell proportions are similar in callus, bone and BM on d5, but there is substantial B cell expansion/recruitment in the callus on d21 (**Figure 4**). Generally, this biphasic immune response in the callus and the differential enrichment/activation of innate vs adaptive immune cells between d5 and d21 explains the reported long-lasting effects of immune cells on the healing process that extends far beyond the initial inflammatory phase (2). On d21, B cell expansion represents the largest immune response to fracture, with a ~450% increase in the proportion of these cells in the callus over resting BM and bone (of d5) (**Figure 4H**). However, on d21, there is also a marked increase in the proportion of B cells in the unfractured bone of mice receiving fracture (**Figure 4H**), indicating a large systemic response that stands in contrast to the localized responses observed in all other immune cell types.

The systemic B cell response likely represents the transport of antigen from the fracture area to secondary lymphoid organs, where a primary or memory B cell response is initiated and spread systemically. B cell responses occur overwhelmingly in an antigen-specific manner, so it is unlikely that this large expansion of B cells occurs without recognition of a cognate antigen. However, the expansion of antigen-specific B cells would represent a very strong response to self-expressed antigen, a potential trigger to subsequent B and antibody-mediated autoimmunity. Notably, the costimulatory molecules that are often associated with full B cell activation are expressed on few B cells in the callus, so it is possible that the B cell expansion we observe following fracture occurs via a non-canonical mechanism. Therefore, further studies are required to understand how this strong, systemic B-cell response does not trigger auto-immunity. A thorough investigation of the function and specificity of B cells triggered by fracture may begin to provide clues about this process, and potential implications for lifelong health, beyond the immediate role of these cells in fracture healing.

We and others have previously described that DIO mice exhibit profound defects in fracture healing, including defective function of skeletal progenitors (24), early resorption of the soft callus, defective callus mineralization, and aberrant structure of collagen fibers (3, 23). DIO mice, as well as obese patients, are also widely accepted to exhibit a hyper-inflammatory state, with higher levels of serum cytokines mainly produced by activated myeloid cells that infiltrate the adipose tissue in high numbers in response to the hypertrophic expansion of adipose tissues (69). However, this systemic inflammatory state does not necessarily reflect the inflammatory environment in a healing tissue, and the immune-cell composition in the healing callus of the DIO mouse has never been characterized. Unlike the situation in adipose tissues, our data show that the myeloid precursor/PMN population, together with the monocyte populations, were proportionally reduced in DIO vs lean mice callus at d5, a primary timepoint of expansion/recruitment and activation of these cell types (**Figures 6A-D**). However, higher proportions of monocytes and macrophages were activated on d5 in the DIO mice than in the lean callus (**Figures 6E, F**; **S21E-J**). We did also observe a very marked increase in the proportion of DCs in the DIO callus relative to the lean callus (**Figure 6H**), and this increase was largely accounted for by recruitment/expansion of cDC2 and, particularly, Mo-DC (**Figures 6I, J**). As cDC2 and Mo-DC share CMP as a common precursor with PMNs, monocytes and macrophages, it is likely that the inflammatory environment in the callus of DIO mice biases development towards the DC lineage, resulting in more DC (**Figures 6H-J**) and less monocytes (**Figures 6C, D**). Further studies are required to elucidate this point.

The larger DC subpopulations in the DIO mice also display enhanced activation (**Figures 6L-O**), raising the possibility that this could lead to enhanced activation of the adaptive immune response in DIO mice. However, on d21, we observe lower proportions of NK cells (**Figure 7A**) and CD4^+^ T cells (**Figure 7B**) and similar proportions of CD8^+^ T cells (**Figure 7C**) and B cells (**Figure 7D**) in the DIO mice when compared to lean animals. Therefore, although there is a larger number of activated DC in the DIO callus, the imbalance we observe towards expansion of Mo-DC (**Figures 6J, K**) may prevent efficient development of a pro-healing lymphocyte response. Mo-DC appear to effectively upregulate MHC-II and costimulatory molecules in the DIO mice (**Figures 6N, O**), so this does not appear to be an obvious defect in the ability to activate naïve or memory T cells. Rather, this may represent a nuanced imprinting of adaptive immune cells after activation, perhaps achieved via the production of modulatory cytokines and other mediators by Mo-DC during activation of precursors. This is most obviously a potential outcome in the B cell compartment, in which a gross analysis shows that similar proportions of B cells exit in the callus of lean and DIO mice on both d5 and d21 (**Figure 7D**). However, closer analysis reveals that B cells in the DIO mice may be stuck at precursor stage of development, and therefore mature B cells, which aid in fracture healing, are lacking in the DIO mice (**Figures 7F–H**). This defect in the adaptive immune response alone, or combined with other alterations in the immune compartment, could account for multiple healing defects observed in DIO animals.

In conclusion, while the two waves of immune-cell infiltration into the callus have been previously reported, the results we are showing here characterize the stoichiometry and activation status of individual innate and adaptive immune-cell populations in the healing callus during each wave and identify cells that are specifically enriched in the callus as compared to unfractured bone and BM. Importantly, our data also recognize callus-specific enrichment of particular subsets of DC (Mo-DC and cDC2) during the inflammatory phase as well as a previously undescribed systemic B-cell response during the late repair and remodelling phases. Furthermore, our data underscore pronounced defetcs in the adaptive immune response during the late phases of healing in DIO mice, which migh aid in developing immunomodulatory agents to treat delayed healing and non-unions in diabetic patients.

## Materials and methods

### Animals

Lean and DIO, male C57BL/6J mice were purchased from The Jackson Laboratory (Bar Harbor, ME, USA) at the age of 18 weeks (high-fat diet feeding of DIO mice started at the age of 6 weeks). Mice were allowed to acclimate to the care facility for 2 weeks, during which DIO mice were placed on high-fat diet (60 kcal % fat; Open Source Diets, Research Diets Inc.), while the lean controls received lean diet (10 kcal % fat; Open Source Diets, Research Diets Inc.). At the end of the 2-week acclimation period, obesity was confirmed, and the body weight of DIO mice was significantly higher than that of lean mice (**Figure S25**). The animals were provided with ad libitum access to pelleted feed and water, and they were housed in a 12-hour light/dark cycle at 21.1°C to 22.8°C and 30% to 70% humidity. All animal protocols were approved by the University Committee on Animal Resources (IACUC) at the Pennsylvania State University College of Medicine. Notably, only male mice were used as female mice are more resistant to the development of obesity-associated metabolic changes (70, 71).

### Mid-diaphysis tibial fracture surgery

Open tibial fracture osteotomy was induced in the right hindlimb according to the established protocol that we described previously (3, 26). Fractures were stabilized using an intramedullary nail, and stabilization of the fracture site was confirmed by taking X-ray images post-operatively and at the harvest time using UltraFocus DXA system (Faxitron Bioptics). Harvest timepoints were decided based on studies published by us and others on the healing time course of this fracture model (3, 23, 26). Mice were kept on the corresponding lean or high-fat diet until harvest time.

### IF and histological staining

The fractured limb was harvested from the mid-femur to the tibiotalar joint to avoid disruption of the healing callus. Most of the surrounding soft tissues were removed, and the isolated tissue was fixed in 10% (v/v) neutral buffered formalin (Thermo Fisher Scientific). The intramedullary pin was removed, and the fixed tissue was then decalcified in 14% w/v EDTA tetrasodium (Thermo Fisher Scientific) and embedded in paraffin. Sagittal sections with 5-µm thicknesses spanning the center of the callus were collected. The sections were stained with Hematoxylin/Safranin-O/Fast Green or subjected to IF staining as we described previously (3, 26). Antibodies used in IF staining are listed in **Table S24**.

### RNA extraction and sequencing

Soft tissues surrounding the healing callus were completely removed, and the callus was harvested, avoiding the surrounding intact bone, and immediately snap frozen in liquid nitrogen. RNA was extracted according to the protocol we detailed previously (72). RNA-seq was performed as we described previously (26, 73) using three biological replicates (i.e., three callus tissues). Briefly, the Illumina^®^ Stranded mRNA Prep and Ligation kit (Illumina) was used to prepare cDNA libraries as follows. Oligo (dT) beads were used to purify poly(A) RNA from 200 ng total RNA, and the purified fraction was subjected to fragmentation and reverse transcription, followed by end repair, 3′ adenylation, and adaptor ligation. The unique dual index sequences (IDT^®^ for Illumina^®^ RNA UD Indexes Set A, Ligation, Illumina) were incorporated in the adaptors for multiplexed high-throughput sequencing. PCR amplification and SPRI bead purification (Beckman Coulter) were then performed. Size distribution and concentration of the final product were assessed using BioAnalyzer High Sensitivity DNA Kit (Agilent Technologies). Pooled libraries were diluted to 3 nM using 10 mM Tris-HCl (pH 8.5), denatured, loaded onto an S1 flow cell (Illumina NovaSeq 6000), and run for 2x53 cycles.

### Analysis of RNA-seq data

RNA-seq data were analyzed as we described previously (26). Briefly, Illumina bcl2fastq (released version 2.20.0.422) was used to generate De-multiplexed and adapter-trimmed sequencing reads, allowing no mismatches in the index read. Low quality sequences were trimmed/filtered using BBDuk (ourceforge.net/projects/bbmap/) and the “qtrim=lr trimq=10 maq=10” option. HISAT2 (version 2.1.0) was used to align the filtered reads to the mouse reference genome (GRCm38), applying –no-mixed and –no-discordant options. HTSeq was used to calculate read counts, and DESeq2 was used to determine differentially expressed genes.

For analysis of gene expression profile over the time course, we first applied a generalized linear model (GLM) to raw read counts fitted with a negative binomial distribution and obtained TMM (trimmed mean of M-values)-normalized read counts using an R package TCC (74). Another R package maSigPro (75) was then used to identify differentially expressed genes over the time course between the lean and DIO groups. maSigPro applies linear regression to model gene expression and selects differentially expressed genes. Using Polynomial degree=2 and Q=0.05 as significance cutoffs, we identified the differentially expressed genes between the lean and DIO groups. For GO analysis, the integrated Differential Expression and Pathway analysis (iDEP) (76) and ShinyGO (77) were used, and heatmaps were prepared using iDEP.

### RT-qPCR

cDNA was prepared using ~ 1 μg RNA and SuperScript IV Reverse Transcriptase (Thermo Fisher Scientific). qPCR was performed using TaqMan Fast Advanced Master Mix and TaqMan Gene-Expression Assays (Thermo Fisher Scientific).

### Flow cytometry analysis

The mice were sacrificed, and the healing hematoma of d5 was collected or the healing callus of d21 was shaved. The surrounding cortical bone was avoided. The contralateral, unfractured tibia was also collected from each mouse, the BM was completely flushed out, and each tissue was processed and analyzed separately. The soft tissues were completely removed from the calli and bones. The harvested tissues were minced and digested for 1 h at 37° C in a mixture of collagenase/dispase (1 µg/ml, catalog: 10269638001, Roche) and collagenase D (3 mg/ml, catalog: 11088882001, Roche). The red blood cells were then lysed using 1X RBC lysis buffer (Catalog # 00-4333-57, eBioscience) according to the manufacturer’s protocol. The cells were counted, blocked using 24G2 hybridoma and 20% serum, and stained using the antibodies described in **Table S24**. Isotype controls and fluorescence-minus-one (FMO) controls were employed. The cells were sorted using 23-color BD FACS Symphony (BD Biosciences, San Jose, CA), and the results were analyzed using FlowJo Software 10.6.2 (Treestar, Ashland, OR). The experiment was repeated for 3 biological replicates, and 5 calli were pooled for each replicate.

### Statistical analysis

ANOVA (followed by Tukey’s *post hoc* test) was used to determine statistical significance among three or more groups. Statistical analyses were performed using the GraphPad Prism software. The following symbols were used to indicate significance: (ns) *P* ≥ 0.05; (*) *P* < 0.05; (**) *P* < 0.01; (***) *P* < 0.001.

## Data availability statement

The datasets presented in this study can be found in online repositories. The names of the repository/repositories and accession number(s) can be found below: GSE240390 (GEO- https://www.ncbi.nlm.nih.gov/geo/query/acc.cgi?acc=GSE240390).

## Ethics statement

The animal study was approved by University Committee on Animal Resources (IACUC) at the Pennsylvania State University College of Medicine. The study was conducted in accordance with the local legislation and institutional requirements.

## Author contributions

CN and RE contributed to conception and design of the study. DK and IR generated the data. All authors contributed to data analysis and interpretation. CN and RE wrote the first draft of the manuscript. All authors contributed to the article and approved the submitted version.

## Supplementary material

Supplementary Figure 1
**(A-D, left)** IF co-staining of Col I (red) and Col II (green). DAPI stains nuclei (blue). White arrows in each panel point to the fracture line. The dashed white line outlines the callus. The images show the full callus area from which panels in **Figures 1A-D** were cropped. The scale bar = 500 µm. **(A-D, right)** Safranin O/fast green (Saf-O) staining of the same sections shown in the left panels. The reddish orange staining denotes the extracellular matrix of the soft callus. The scale bar = 500 µm.

Supplementary Figure 2 Principal component analysis (PCA) of the RNA-seq data generated from the callus tissues harvested from lean mice at the indicated timepoints. N = 3 mice at each timepoint.

Supplementary Figure 3
**(A)** Heatmap of significantly (*P_adj_* < 0.05) upregulated (red) and downregulated (blue) genes in the callus of lean mice on d5 relative to d3 (see also **Table S1**). **(B)** GO analysis of significantly upregulated genes on d5 vs d3 (see **Table S2** for the full list of pathways). The *P*adj (-log10) of enrichment is given for each pathway.

Supplementary Figure 4
**(A)** Heatmap of significantly (*P_adj_* < 0.05) upregulated (red) and downregulated (blue) genes in the callus of lean mice on d7 relative to d3 (see also **Table S1**). **(B)** GO analysis of significantly upregulated (red) and downregulated (blue) genes on d7 vs d3 (see **Table S3** for the full list of pathways). The Padj (-log10) of enrichment is given for each pathway.

Supplementary Figure 5
**(A)** Heatmap of significantly (*P_adj_* < 0.05) upgregulated (red) and downregulated (blue) genes in the callus of the lean mice on d10 relative to d7 (see also **Table S1**). **(B)** GO analysis of significantly upregulated (red) and downregulated (blue) on d10 vs d7 (see **Table S4** for the full list of pathways.) The *P*adj (-log10) of enrichment is given for each pathway.

Supplementary Figure 6
**(A)** Heatmap of significantly (*P_adj_* < 0.05) upregulated (red) and downregulated (blue) genes in the callus of lean mice on d14 relative to d10 (see also **Table S1**). **(B)** GO analysis of significantly downregulated genes on d14 vs d10 (no KEGG pathway enrichment was detected for genes that were upregulated on d14 vs d10) (see **Table S5** for the full list of pathways). The *P*adj (-log10) of enrichment is given for each pathway.

Supplementary Figure 7
**(A)** Heatmap of significantly (*P_adj_* < 0.05) upregulated (red) and downregulated (blue) genes in the callus of lean mice on d21 relative to d14 (see also **Table S1**). **(B)** GO analysis of significantly upregulated (red) and downregulated (blue) genes on d21 vs d14 (see **Table S7** for the full list of pathways). The Padj (-log10) of enrichment is given for each pathway. **(C)** Presentation of the RNA-seq data showing the expression pattern of CD45 mRNA (a general immune-cell marker). The results indicate a sharp increase in immune-cell response between days 14 and 21.

Supplementary Figure 8 Single cell populations liberated from marrow, bone or callus were analyzed by FC using the gating strategies outlined in **Table S8**. Quantification **(A)** and assessment of activation **(B, C)** of bulk monocytes. Activation of monocyte populations was assessed by measuring the proportion of singlets expressing the costimulatory molecules CD86 **(B)** and MHC Class II **(C)**. N = 3 for all analyzed tissues. The bar graphs present average ± SEM. (*) P < 0.05 and (***) P < 0.001 using one-way ANOVA followed by Turkey's post hoc test.

Supplementary Figure 9 Single cell populations liberated from marrow, bone or callus were analyzed by FC using the gating strategies outlined in **Table S8** and **Figure 2E**. Activation of macrophage populations was assessed by measuring the proportion of singlets expressing MHC Class II **(A)**, and the costimulatory melecules CD40 **(B)** and CD86 **(C)**. N = 3 for all analyzed tissues. The bar graphs present average ± SEM. (*) P < 0.05. (**) P < 0.01. and (***) P < 0.001 using one-way ANOVA followed by Tukey's post hoe test.

Supplementary Figure 10 Single cell populations liberated from marrow. bone or callus were analyzed by FC using the gating strategies outlined in **Table S8**. Activation of pDC populations was assessed by measuring the proportion of singlets expressing MHC Class II **(A)**. the cell surface levels of MHC Class II **(B)**. and the proportion of singlets expressing the costimulatory molecules CD40 **(C)**. N = 3 for all analvzed tissues. The bar graphs present average ± SEM. (*) P < 0.05. (**) P < 0.01. and (***) P < 0.001 using one-way ANOVA followed by Tukey's post hoc test.

Supplementary Figure 11 Single cell populations liberated from marrow, bone or callus were analyzed by FC using the gating strategies outlined in **Table S8**. Activation of cDC2 populations was assessed by measuring the cell surface levels of MHC Class II **(A)** and the proportion of singlets expressing the costimulatory molecules CD40 **(B)** and GITRL **(C)**. N = 3 for all analyzed tissues. The bar graphs present average ± SEM. (***) P < 0.001 using one-way ANOVA followed by Tukey's post hoc test.

Supplementary Figure 12 Single cell populations liberated from marrow, bone or callus were analyzed by FC using the gating strategies outlined in **Table S8**. Activation of Mo-DC populations was assessed by measuring the proportion of singlets expressing the costimulatory molecules CD40 **(A)** and GITRL **(B)** and the cell surface levels of MHC Class II **(C)**. **(D)** RT-qPCR quantitation of Cxcl16 mRNA using total-callus RNA isolated from lean mice at the indicated timepoints. The level of Cxcl16 mRNA was normalized to that of B-actin mRNA, and the normalized level on d3 is defined as 1. N = 3 for FC analysis **(A-C)**. and N = 4-5 for RT-qPCR analysis **(D)**. The bar graphs **(A-C)** and dots **(D)** present average ± SEM. (*) P < 0.05. (**) P < 0.01, and (***) P< 0.001 using one-way ANOVA followed by Tukey's post hoc test.

Supplementary Figure 13 Single cell populations liberated from marrow. bone or callus were analyzed by FC using the gating strategies outlined in **Table S8**. Quantification of **(A)** NK T cells, and **(B)** CD4- CD8-T cells. Activation of B cells (identified and quantified in **Figures 4G, H**) was assessed by measuring the proportion of singlets expressing the costimulatory molecule CD86 **(C)**. **(D-F)** RT-qPCR quantitation of Ccl5 **(D)**. Ifng **(E)**. and Tnf **(F)** mRNAs using total-callus RNA isolated from lean mice at the indicated timepoints. The level of each transcript was normalized to that of B-actin mRNA. and the normalized level on d3 is defined as I. N= 3 or 6 for FC analysis **(A-C)**. and N = 4-5 for RT-qPCR analysis **(D-F)**. The bar graphs **(A-C)** and dots **(D-F)** present average ± SEM.(*) P<0.05. (**) P< 0.01.(***) P = 0.001. and (****) P < 0.0001 using one-way ANOVA followed by Tukey's post hoc test.

Supplementary Figure 14
**(A-D, left)** IF co-staining of Col I (red) and Col II (green) . DAPI stains nuclei (blue). The figures are the same shown in **Figures 5A-D**. **(A-D,** right**)** Saf-O staining of the sections shown in the left panels. The reddish orange staining denotes the extracellular matrix of the soft callus.The scale bar = 500 µm.

Supplementary Figure 15 Heatmap of significantly (*P_adj_* < 0.05) upregulated (red) and downregulated (blue) genes in the callus of DIO mice relative to the callus of lean mice on d3 (**Table S11**). **(B)** GO analysis of significantly downregulated genes in the callus of DIO mice relative to the callus of lean mice (d3) (see **Table S12** for the full list of pathways). The *P*adj (-log10) of enrichment is given for each pathway. No pathway enrichment was detected for significantly upregulated genes.

Supplementary Figure 16 Heatmap of significantly (*P_adj_* < 0.05) upregulated (red) and downregulated (blue) genes in the callus of DIO mice relative to the callus of lean mice on d5 (**Table S13**). **(B)** GO analysis of significantly upregulated (red) and downregulated (blue) genes in the callus of DIO mice relative to the callus of lean mice (d5) (see **Table S14** for the full list of pathways). The *P*adj (-log 10) of enrichment is given for each pathway.

Supplementary Figure 17 Heatmap of significantly (*P_adj_* < 0.05) upregulated (red) and downregulated (blue) genes in the callus of DIO mice relative to the callus of lean mice on d7 (**Table S15**). **(B)** GO analysis of significantly upregulated (red) and downregulated (blue) genes in the callus of DIO mice relative to the callus of lean mice (d7) (see **Table S16** for the full list of pathways). The *P*adj (-log I0) of enrichment is given for each pathway.

Supplementary Figure 18 Heatmap of significantly (*P_adj_* < 0.05) upregulated (red) and downregulated (blue) genes in the callus of DIO mice relative to the callus of lean mice on d10 (**Table S17**). **(B)** GO analysis of significantly upregulated (red) and downregulated (blue) genes in the callus of DIO mice relative to the callus of lean mice (d10) (see **Tables S18**, **S19** for the full list of pathways). The *P*adj (-log10) of enrichment is given for each pathway. **(C)** Presentation of the RNA-seq data showing the expression pattern of Acan and Col2a1 mRNAs (both are chondrocyte markers) in the callus of lean (red) and DIO (green) mice at the indicated timepoints. The results indicate early resorption of the soft callus in DIO mice.

Supplementary Figure 19 Heatmap of significantly (*P_adj_* < 0.05) upregulated (red) and downregulated (blue) genes in the callus of DIO mice relative to the callus of lean mice on d14 (**Table S20**). **(B)** GO analysis of significantly upregulated (red) and downregulated (blue) genes in the callus of DIO mice relative to the callus of lean mice (d14) (see **Table S21** for the full list of pathways). The *P*adj (-log 10) of enrichment is given for each pathway.

Supplementary Figure 20 Heatmap of significantly (*P_adj_* < 0.05) upregulated (red) and downregulated (blue) genes in the callus of DIO mice relative to the callus of lean mice on d21 (**Table S22**). **(B)** GO analysis of significantly upregulated (red) and downregulated (blue) genes in the callus of DIO mice relative to the callus of lean mice (d21) (see **Table S23** for the full list of pathways). The Padj (-log10) of enrichment is given for each pathway.

Supplementary Figure 21 Single cell populations liberated from the fracture callus were analyzed by FC using the gating strategies outlined in **Table S8** and shown in **Figures 2A** and **6A** (PMNs. classical and non-classical monocytes). Quantification of **(A)** total monocytes and **(D)** macrophages. Activation of myeloid cell populations was assessed by measuring the proportion of singlets expressing MHC Class II **(C, F)** and the costimulatory molecules CD86 **(B, I)**. CD40 (G). CD80 **(H)**. and GITRL **(J)** and by measuring the cell surface levels of MHC Class II **(E)**. The bar graphs present average ± SEM. (*) P < 0.05. (**) P < 0.01. and (***) P < 0.001 using one-way ANOVA followed by Tukey's post hoc test.

Supplementary Figure 22 Single cell populations liberated from the fracture callus were analyzed by FC using the gating strategies outlined in **Table S8**. Quantification of **(A)** pDC and **(B)** cDC1 in the callus of lean and DIO mice. Activation of pDC was assessed by measuring the proportion of singlets expressing MHC Class I1 **(C)** and the costimulatory molecule CD40 **(D)** and by measuring the cell surface levels of MHC Class II **(E)**. **(F)** RT-qPCR quantitation of Cxcl16 mRNA using total-callus RNA isolated from lean or DIO mice at the indicated timepoints. The level of Cxcli6 mRNA was normalized to that of B-actin mRNA, and the normalized level on d3 in lean mice is defined as 1. N = 3 for FC analysis **(A-E)**, and N = 4-5 for RT-qPCR analysis **(F)**. The bar graphs present average ± SEM. (*) P < 0.05, (**) P < 0.01, (***) P < 0.001, and (****) P < 0.0001 using one-way ANOVA followed by Tukey's post hoc test.

Supplementary Figure 23 Single cell populations liberated from the fracture callus were analyzed by FC using the gating strategies outlined in **Table S8** and **Figures 4A, D**. Quantification of **(A)** NK T cells and **(B)** CD4-CD8- T cells. **(C-E)** RT-qPCR quantitation of Cel5 **(C)**. Ifng **(D)**. and Tnf **(E)** mRNAs using total-callus RNA isolated from lean or DIO mice at the indicated timepoints. The level of each transcript was normalized to that of B-actin mRNA. and the normalized level on d3 in lean mice is delined as 1. N = 3 for FC analysis **(A, B)**. and N = 4-5 for RI-qPCR analysis **(C-E)**. The bar graphs present average ± SEM. (*) P < 0.05. (**) P < 0.01. (***) P < 0.001. and (****) P < 0.0001 using one-way ANOVA followed by Tukey's post hoc test.

Supplementary Figure 24 Presentation of the RNA-seq data showing the expression pattern of L.ck and Cd3e mRNAs (both genes are involved in T-cell activation) in the callus of lean mice at the indicated timepoints.

Supplementary Figure 25 Body weight (in grams "g") of all lean and DIO mice used in the RNA-seg experiment at the time they underwent fracture surgery. N = 18 in each group, which corresponds to 6 timepoints and 3 biological replicates in each. The bar graphs present average ± SEM. (****) P <0.0001 using Student's t-test.
