# Supplementary material for: Distinct defects in early innate and late adaptive immune responses typify impaired fracture healing in diet-induced obesity
Source: Front Immunol. 2023 Oct 3;14:1250309. doi: 10.3389/fimmu.2023.1250309 (PMC10579581; doi:10.3389/fimmu.2023.1250309)
Supplement: Supplementary file 2 [file Table_1.docx]

**Table S8. Phenotype of immune cells**

| **Cell Type** | **Cell Subsets** | **Phenotype** |
| --- | --- | --- |
| Immune Cells |  | CD45^+^ |
| Polymorphonuclear granulocytes (PMNs) and myeloid cell progenitors |  | CD45^+^, CD3^-^, CD19^-^, NK1.1^-^, CD11b^+^, F4/80^-^, Ly6C^+^, Ly6G^+^ |
| Macrophages |  | CD45^+^, CD3^-^, CD19^-^, NK1.1^-^, CD11b^+^, F4/80^+^, Ly6C^-^ |
| Monocytes |  | CD45^+^, CD3^-^, CD19^-^, NK1.1^-^, CD11b^+^, F4/80^-^, Ly6G^-^, Ly6C^+^ |
|  | Non-Classical Monocytes | CD45^+^, CD3^-^, CD19^-^, NK1.1^-^, CD11b^+^, F4/80^+^, Ly6G^-^, Ly6C^Lo^ |
|  | Classical Monocytes | CD45^+^, CD3^-^, CD19^-^, NK1.1^-^, CD11b^+^, F4/80^+^, Ly6G^-^, Ly6C^Hi^ |
| Bulk Dendritic cells (DC) |  | CD45^+^, CD3^-^, CD19^-^, NK1.1^-^, F4/80^-^, CD11c^+^ |
|  | Conventional DC 1 (cDC1) | CD45^+^, CD3^-^, CD19^-^, NK1.1^-^, F4/80^-^, CD11c^+^, B220^-^, Ly6C^-^, CD11b^-^, CD8α^+^ |
|  | Conventional DC 2 (cDC2) | CD45^+^, CD3^-^, CD19^-^, NK1.1^-^, F4/80^-^, CD11c^+^, B220^-^, Ly6C^-^, CD11b^+^, CD8α^-^ |
|  | Plasmacytoid DC (pDC) | CD45^+^, CD3^-^, CD19^-^, NK1.1^-^, F4/80^-^, CD11c^+^, B220^+^ |
|  | Monocyte-Derived DC (Mo-DC) | CD45^+^, CD3^-^, CD19^-^, NK1.1^-^, F4/80^-^, CD11c^+^, B220^-^, Ly6C^+^ |
| NK Cells |  | CD45^+^, CD19^-^, NK1.1^+^, CD3^-^ |
| NK T cells |  | CD45^+^, CD19^-^, NK1.1^+^, CD3^+^ |
| Bulk T cells |  | CD45^+^, CD19^-^, NK1.1^-^, CD3^+^ |
|  | CD4^+^ T cells | CD45^+^, CD19^-^, NK1.1^-^, CD3^+^, CD4^+^, CD8^-^ |
|  | CD8^+^ T cells | CD45^+^, CD19^-^, NK1.1^-^, CD3^+^, CD4^-^, CD8^+^ |
|  | CD4^-^ CD8^-^ T cells | CD45^+^, CD19^-^, NK1.1^-^, CD3^+^, CD4^-^, CD8^-^ |
| B cells |  | CD45^+^, NK1.1^-^, CD3^-^, CD19^+^ |
|  | Mature B cells | CD45^+^, NK1.1^-^, CD3^-^, CD19^+^, B220^Hi^ |
|  | Immature (Pro?) B cells | CD45^+^, NK1.1^-^, CD3^-^, CD19^+^, B220^Lo^ |
