# Supplementary material for: Distinct defects in early innate and late adaptive immune responses typify impaired fracture healing in diet-induced obesity
Source: Front Immunol. 2023 Oct 3;14:1250309. doi: 10.3389/fimmu.2023.1250309 (PMC10579581; doi:10.3389/fimmu.2023.1250309)
Supplement: Supplementary file 3 [file Table_2.docx]

**Table S24. List of antibodies**

**IF staining**

| **Protein** | **Primary antibody** |
| --- | --- |
| Col I | Abcam  (biotinylated primary antibody; ab24821) |
| Col II | Abcam  (ab34712) |
| Col X | Abcam  (ab58632) |

**Flow cytometry**

| **Protein** | **Antibody** |
| --- | --- |
| CD11b | BD Pharmingen™ Alexa Fluor™ 488 Rat Anti-CD11b  557672 |
| CD40 | BD OptiBuild™ BB700 Rat Anti-Mouse CD40  742136 |
| CD4 | Brilliant Violet 421™ anti-mouse CD4 Antibody  100437 |
| GITRL | BD OptiBuild™ BV480 Rat Anti-Mouse GITR Ligand  746507 |
| CD19 | Brilliant Violet 785™ anti-mouse CD19 Antibody  115543 |
| TCR β | BD Pharmingen™ APC Hamster Anti-Mouse TCR β Chain  553174 |
| Ly6C | BD Horizon™ R718 Rat Anti-Mouse Ly-6C  566987 |
| CD11c | APC/Cyanine7 anti-mouse CD11c Antibody  117324 |
| NK-1.1 | BD Horizon™ BUV395 Mouse Anti-Mouse NK-1.1  564144 |
| CD80 | BD OptiBuild™ BUV496 Hamster Anti-Mouse CD80  741091 |
| Ly6G | BD Horizon™ BUV563 Rat Anti-Mouse Ly-6G  612921 |
| F4/80 | BD OptiBuild™ BUV737 Rat Anti-Mouse F4/80  749283 |
| MHC class II | PE anti-mouse I-A/I-E Antibody  107608 |
| CD8a | PE/Cyanine5 anti-mouse CD8a Antibody  100710 |
| CD86 | BD Pharmingen™ PE-Cy™7 Rat Anti-Mouse CD86  560582 |
| CD45R/B220 | BD Horizon™ BV711 Rat Anti-Mouse CD45R/B220  563892 |
| CD45 | BD Horizon™ BV650 Rat Anti-Mouse CD45  563410 |
