# Supplementary material for: Distinct defects in early innate and late adaptive immune responses typify impaired fracture healing in diet-induced obesity
Source: Front Immunol. 2023 Oct 3;14:1250309. doi: 10.3389/fimmu.2023.1250309 (PMC10579581; doi:10.3389/fimmu.2023.1250309)

# Fig. S1

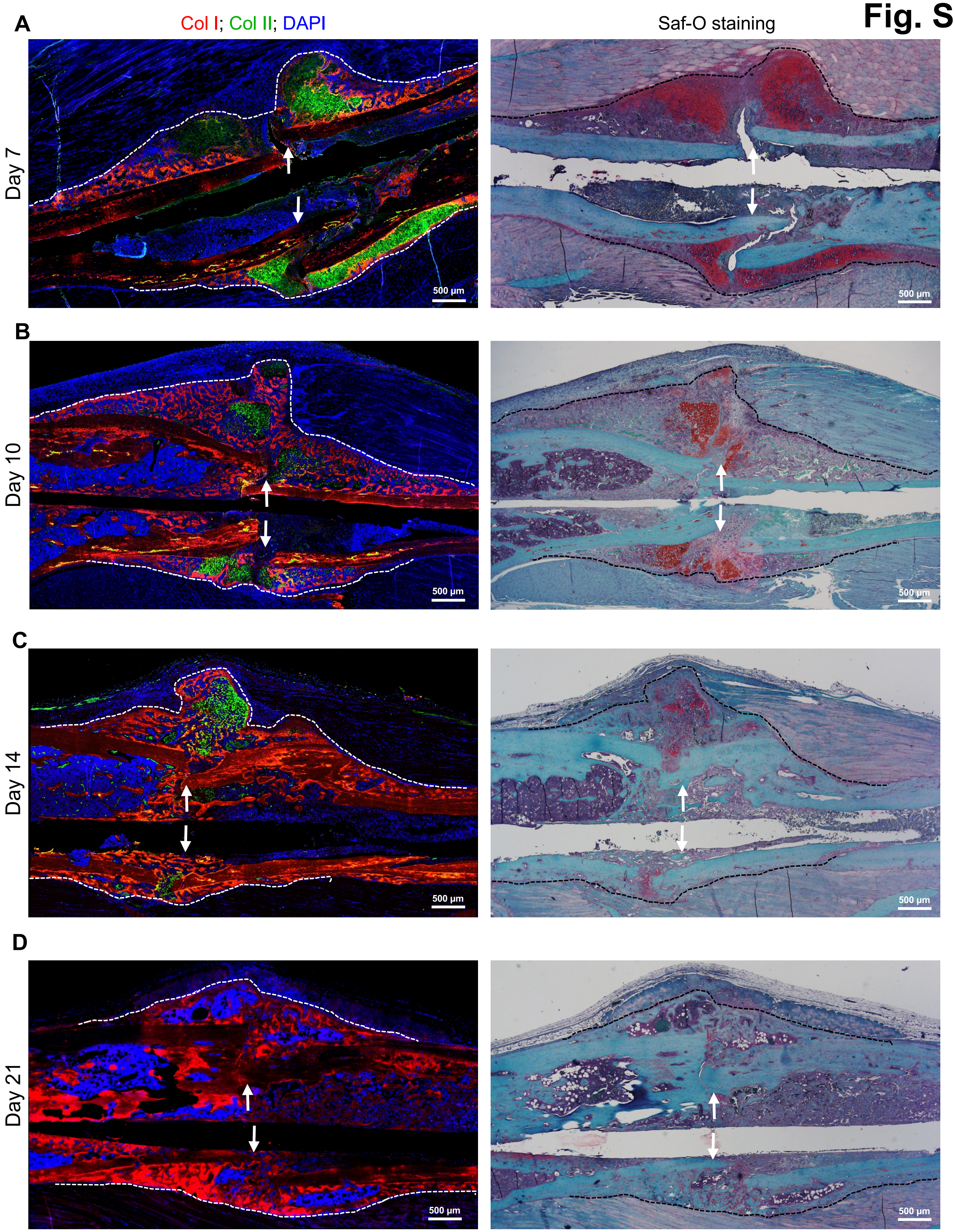
A
Col I; Col II; DAPI
Saf-O staining
Day 7
500 µm
500 µm
B
Day 10
500 µm
500 µm
C
Day 14
500 µm
500 µm
D
Day 21
500 µm
500 µm

**Fig. S2**

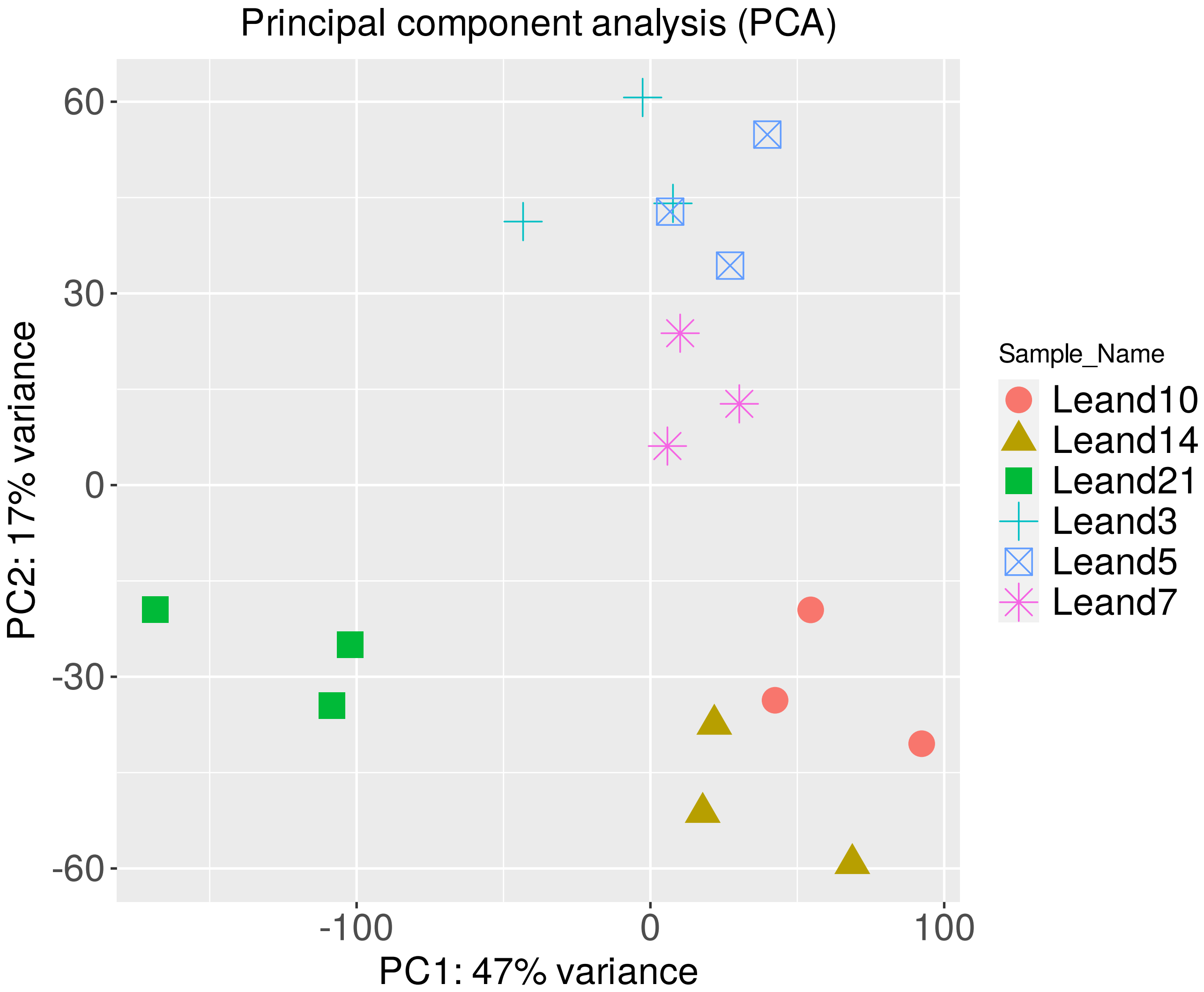
Principal component analysis (PCA)
60
30
0
-30
-60
-100
0
100
PC1: 47% variance
PC2: 17% variance
Sample_Name
Leand10
Leand14
Leand21
Leand3
Leand5
Leand7

**Fig. S3**

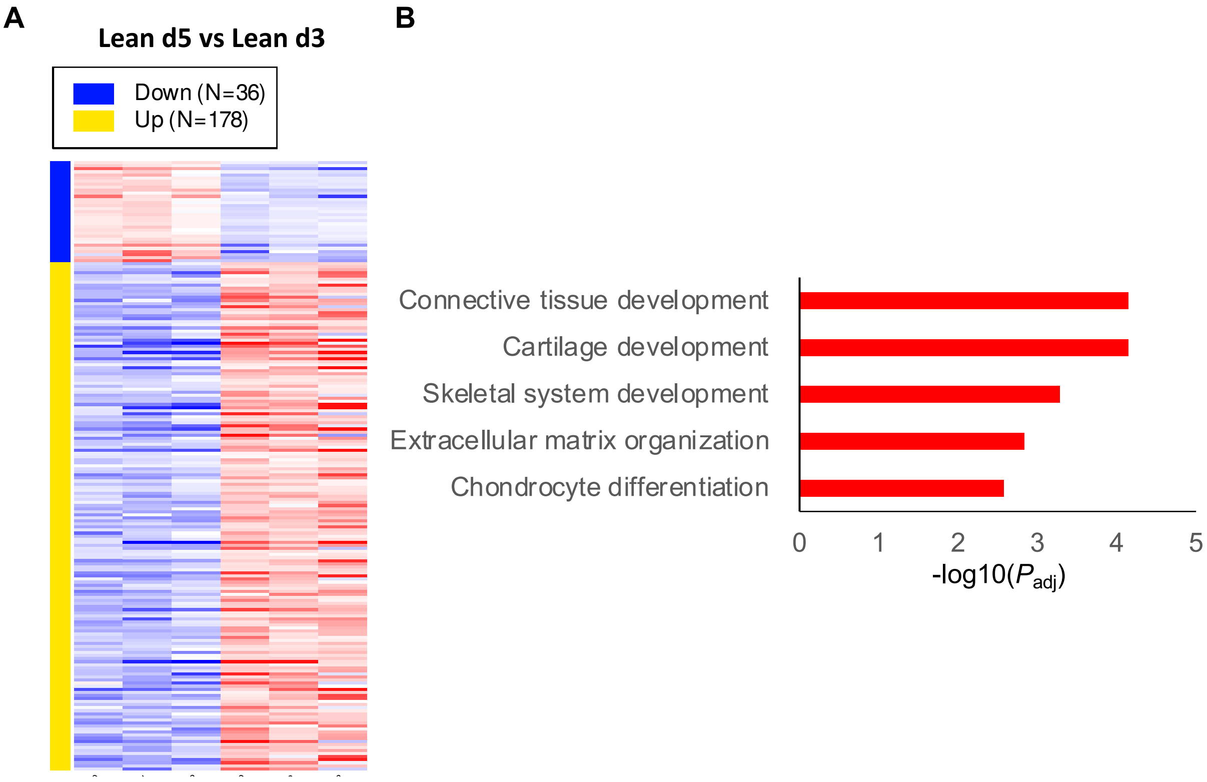
A
Lean d5 vs Lean d3
Down (N=36)
Up (N=178)
B
Connective tissue development
Cartilage development
Skeletal system development
Extracellular matrix organization
Chondrocyte differentiation
0
1
2
3
4
5
-log10($P_{adj}$)

**Fig. S4**

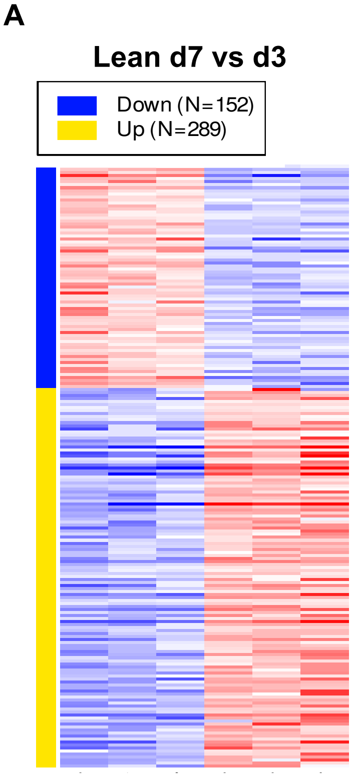
A
Lean d7 vs d3
Down (N=152)
Up (N=289)

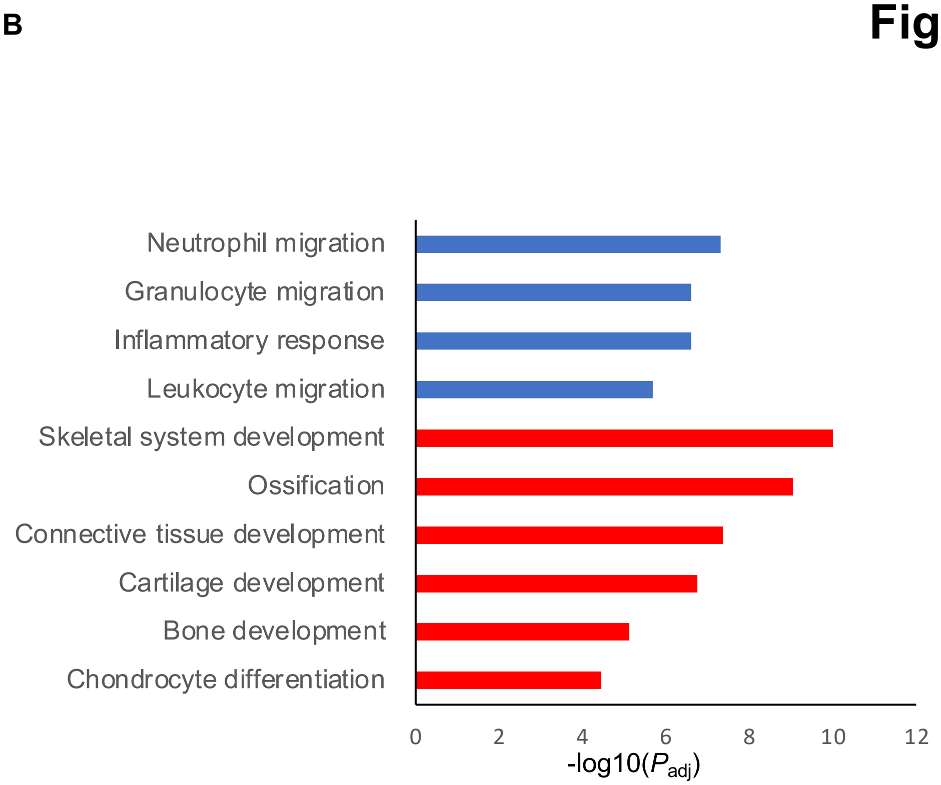
B
Neutrophil migration
Granulocyte migration
Inflammatory response
Leukocyte migration
Skeletal system development
Ossification
Connective tissue development
Cartilage development
Bone development
Chondrocyte differentiation
0
2
4
6
8
10
12
-log10($P_{adj}$)

**Fig. S5**

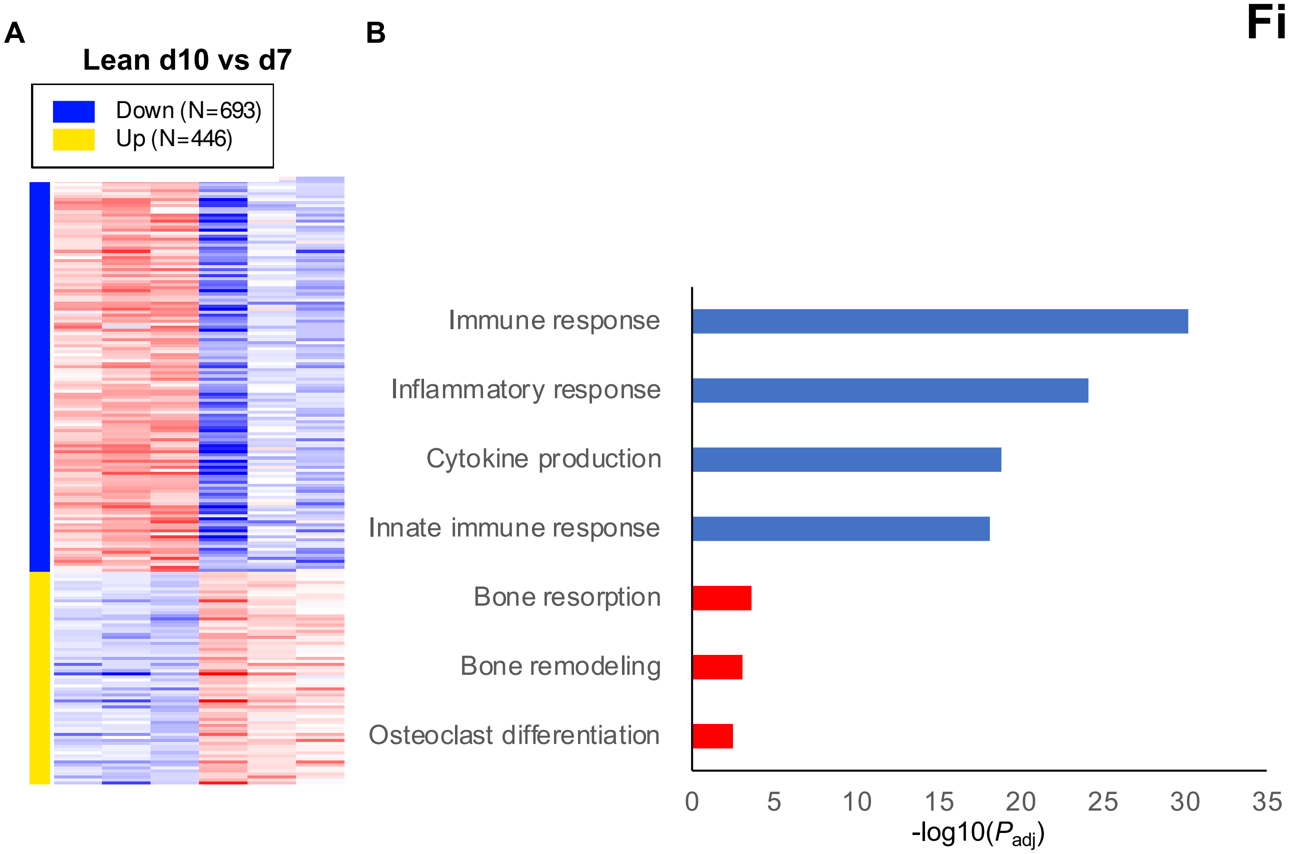
A
Lean d10 vs d7
Down (N=693)
Up (N=446)
B
Immune response
Inflammatory response
Cytokine production
Innate immune response
Bone resorption
Bone remodeling
Osteoclast differentiation
0
5
10
15
20
25
30
35
-log10($P_{adj}$)

Fig. S6

A

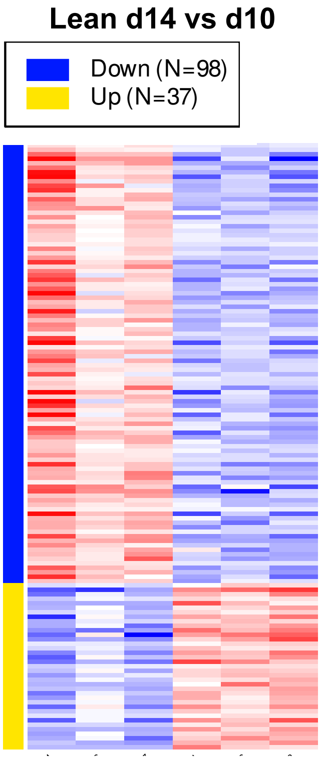
Lean d14 vs d10
Down (N=98)
Up (N=37)

B

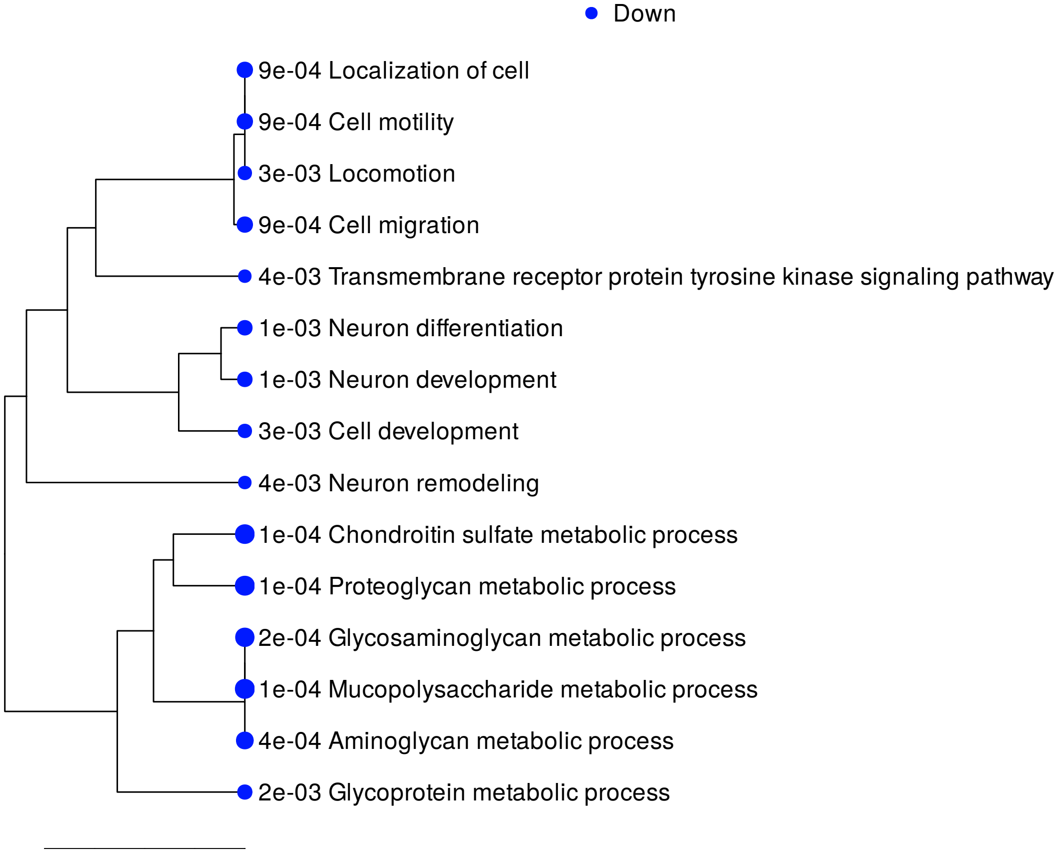
Down
9e-04 Localization of cell
9e-04 Cell motility
3e-03 Locomotion
9e-04 Cell migration
4e-03 Transmembrane receptor protein tyrosine kinase signaling pathway
1e-03 Neuron differentiation
1e-03 Neuron development
3e-03 Cell development
4e-03 Neuron remodeling
1e-04 Chondroitin sulfate metabolic process
1e-04 Proteoglycan metabolic process
2e-04 Glycosaminoglycan metabolic process
1e-04 Mucopolysaccharide metabolic process
4e-04 Aminoglycan metabolic process
2e-03 Glycoprotein metabolic process

# Fig. S7

A

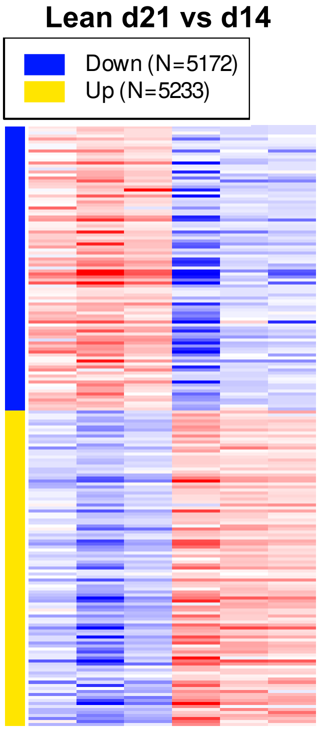
Lean d21 vs d14
Down (N=5172)
Up (N=5233)

B

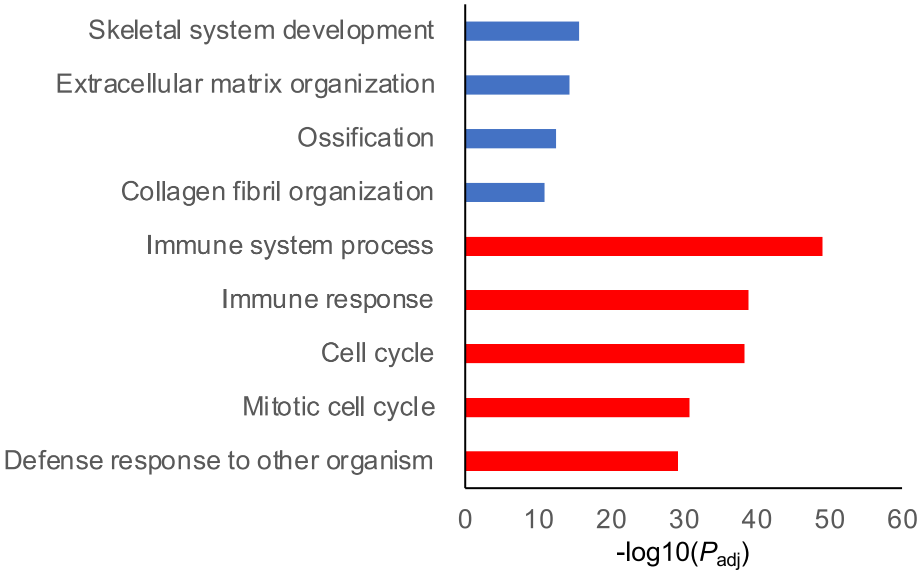
Skeletal system development
Extracellular matrix organization
Ossification
Collagen fibril organization
Immune system process
Immune response
Cell cycle
Mitotic cell cycle
Defense response to other organism
0
10
20
30
40
50
60
-log10(Padj)

C

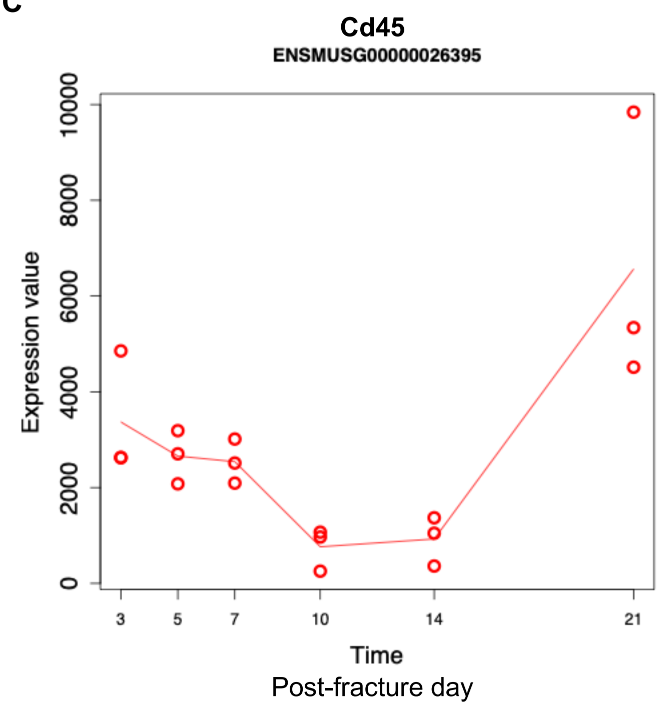
Cd45
ENSMUSG00000026395
Expression value
0
2000
4000
6000
8000
10000
3
5
7
10
14
21
Time
Post-fracture day

# Fig. S8

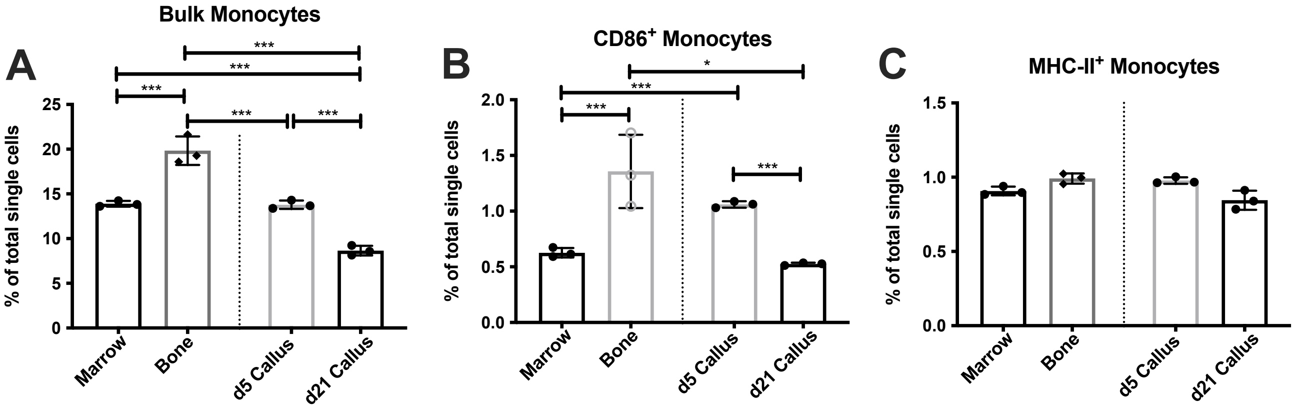
A
Bulk Monocytes
% of total single cells
0
5
10
15
20
25
***
***
***
***
***
Marrow
Bone
d5 Callus
d21 Callus
B
CD86+ Monocytes
% of total single cells
0.0
0.5
1.0
1.5
2.0
***
***
*
***
Marrow
Bone
d5 Callus
d21 Callus
C
MHC-II+ Monocytes
% of total single cells
0.0
0.5
1.0
1.5
Marrow
Bone
d5 Callus
d21 Callus

# Fig. S9

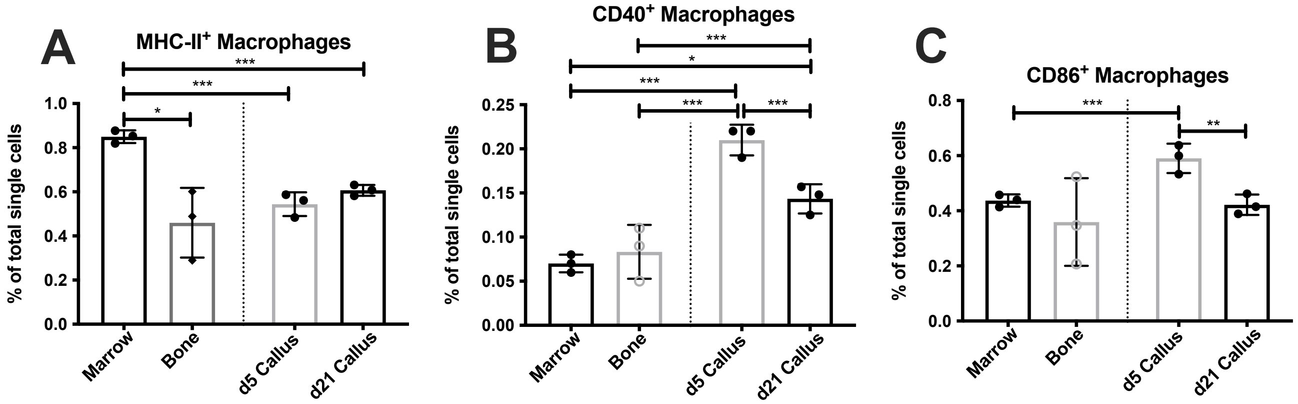
A
MHC-II+ Macrophages
% of total single cells
1.0
0.8
0.6
0.4
0.2
0.0
*
***
***
Marrow
Bone
d5 Callus
d21 Callus
B
CD40+ Macrophages
% of total single cells
0.25
0.20
0.15
0.10
0.05
0.00
***
*
***
***
***
Marrow
Bone
d5 Callus
d21 Callus
C
CD86+ Macrophages
% of total single cells
0.8
0.6
0.4
0.2
0.0
***
**
Marrow
Bone
d5 Callus
d21 Callus

**Fig. S10**

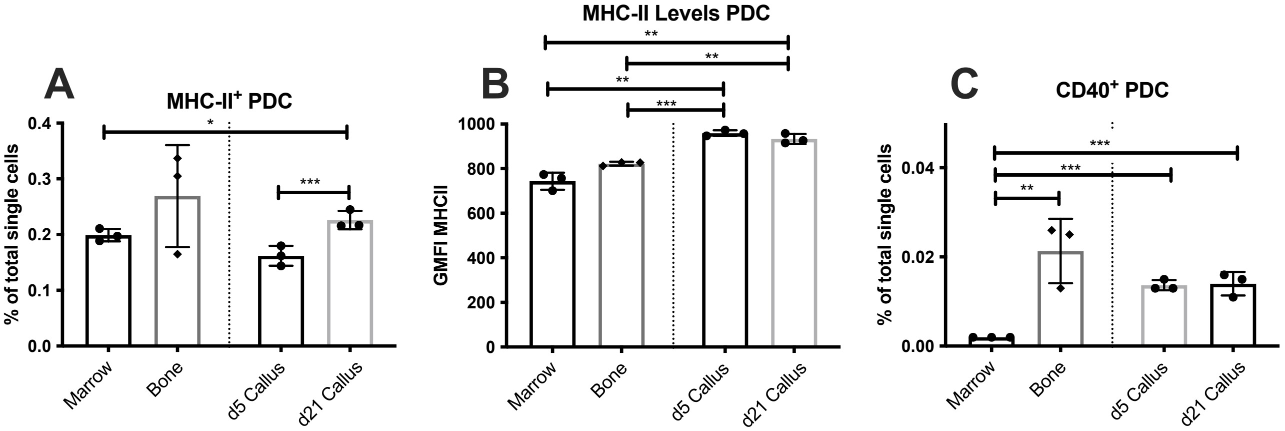
A
MHC-II+ PDC
% of total single cells
0.4
0.3
0.2
0.1
0.0
*
***
Marrow
Bone
d5 Callus
d21 Callus
B
MHC-II Levels PDC
GMFI MHCII
1000
800
600
400
200
0
**
**
**
***
Marrow
Bone
d5 Callus
d21 Callus
C
CD40+ PDC
% of total single cells
0.04
0.02
0.00
***
***
**
Marrow
Bone
d5 Callus
d21 Callus

Fig. S11

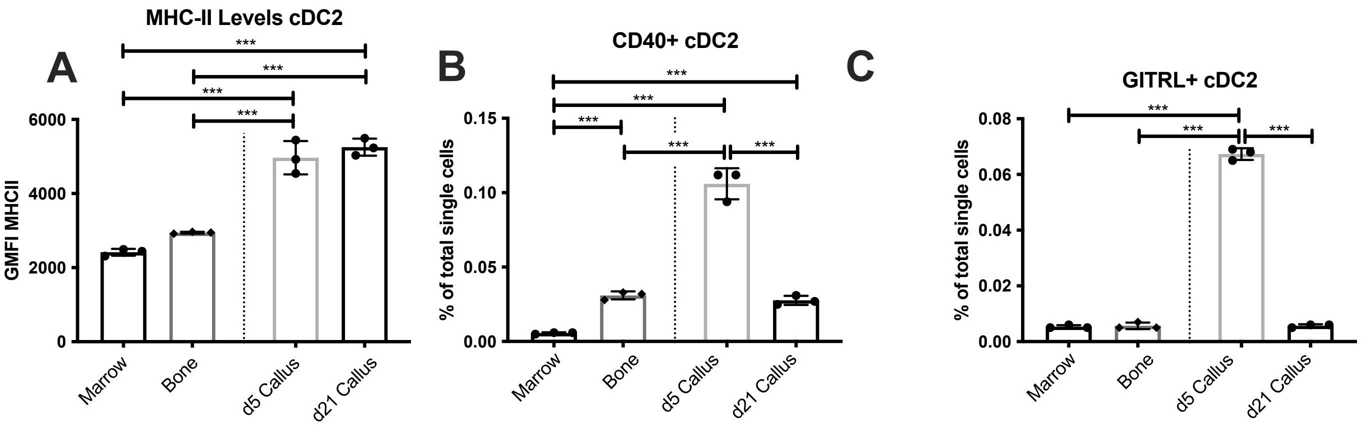
A
MHC-II Levels cDC2
GMFI MHCII
***
6000
4000
2000
0
Marrow
Bone
d5 Callus
d21 Callus
B
CD40+ cDC2
% of total single cells
0.15
0.10
0.05
0.00
C
GITRL+ cDC2
0.08
0.06
0.04
0.02
0.00

# Fig. S12

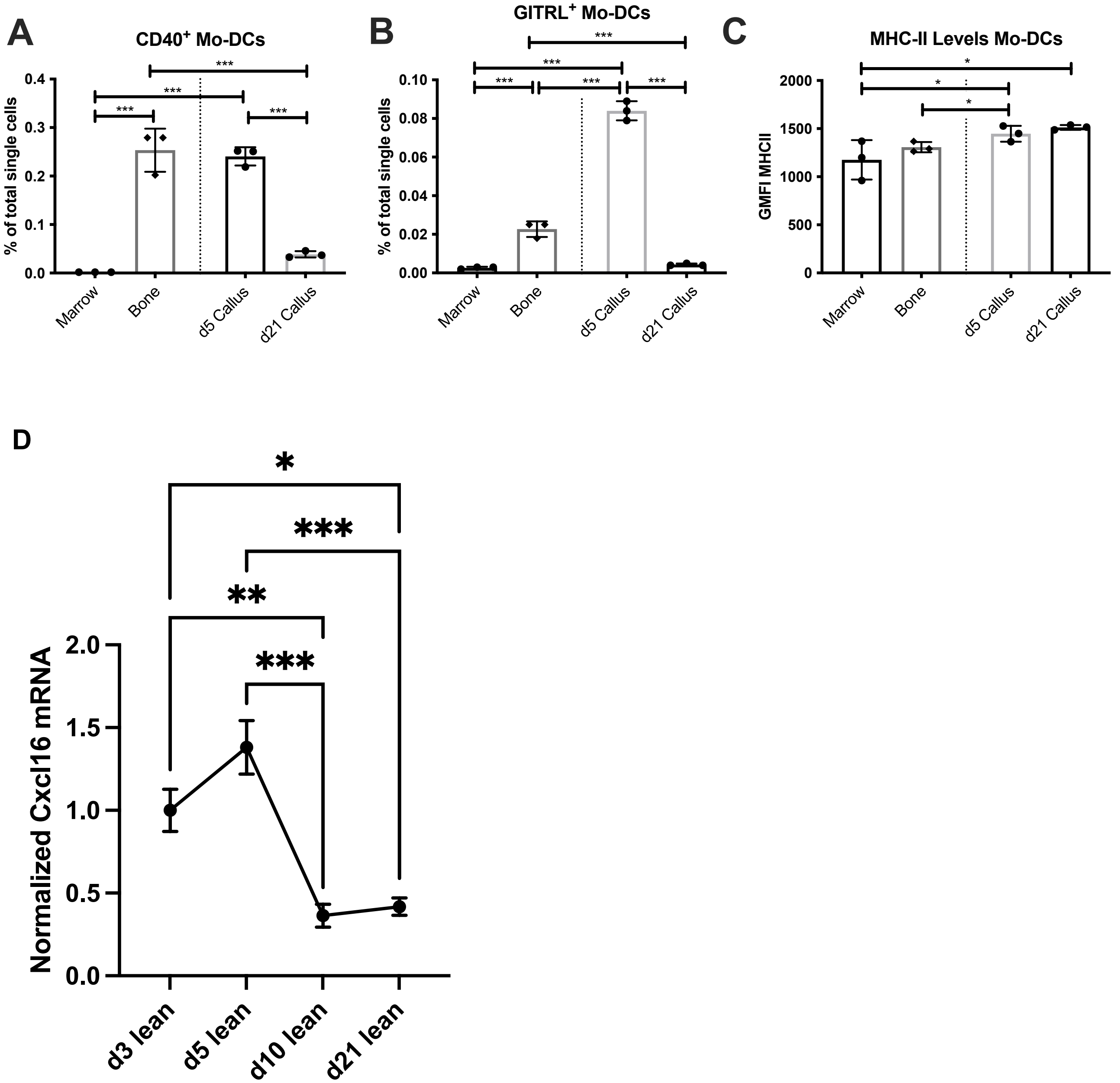
A
CD40+ Mo-DCs
% of total single cells
0.4
0.3
0.2
0.1
0.0
***
***
***
***
Marrow
Bone
d5 Callus
d21 Callus
B
GITRL+ Mo-DCs
% of total single cells
0.10
0.08
0.06
0.04
0.02
0.00
***
***
***
***
***
Marrow
Bone
d5 Callus
d21 Callus
C
MHC-II Levels Mo-DCs
GMFI MHCII
2000
1500
1000
500
0
*
*
*
Marrow
Bone
d5 Callus
d21 Callus
D
*
***
**
***
Normalized Cxcl16 mRNA
2.0
1.5
1.0
0.5
0.0
d3 lean
d5 lean
d10 lean
d21 lean

# Fig. S13

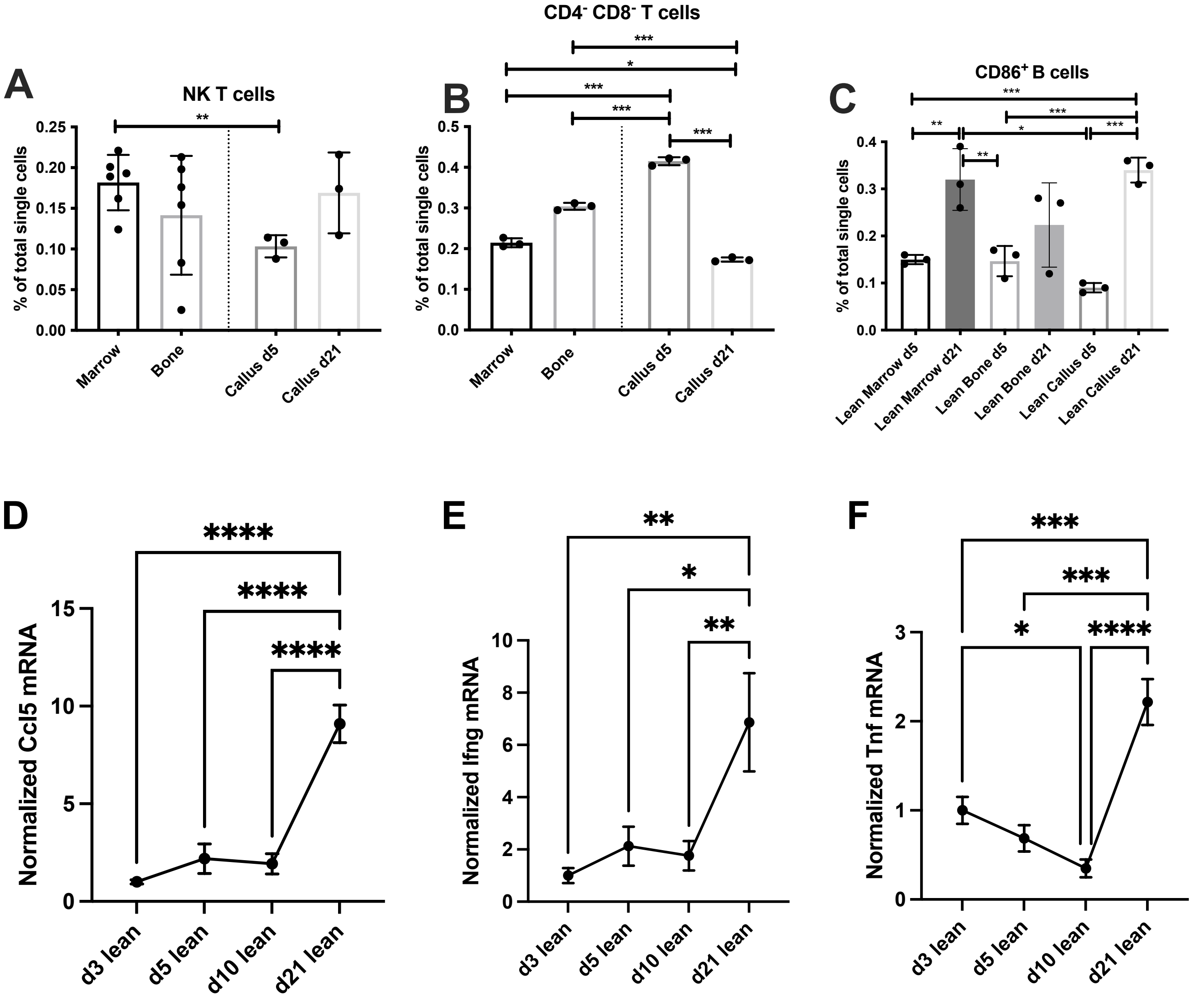

A
NK T cells
% of total single cells
0.25
0.20
0.15
0.10
0.05
0.00
**
Marrow
Bone
Callus d5
Callus d21
B
CD4⁻ CD8⁻ T cells
% of total single cells
0.5
0.4
0.3
0.2
0.1
0.0
***
*
***
***
***
Marrow
Bone
Callus d5
Callus d21
C
CD86⁺ B cells
% of total single cells
0.4
0.3
0.2
0.1
0.0
***
**
***
*
***
**
Lean Marrow d5
Lean Marrow d21
Lean Bone d5
Lean Bone d21
Lean Callus d5
Lean Callus d21
D
Normalized Ccl5 mRNA
15
10
5
0
****
****
****
d3 lean
d5 lean
d10 lean
d21 lean
E
Normalized Ifng mRNA
10
8
6
4
2
0
**
*
**
d3 lean
d5 lean
d10 lean
d21 lean
F
Normalized Tnf mRNA
3
2
1
0
***
***
*
****
d3 lean
d5 lean
d10 lean
d21 lean

# Fig. S14

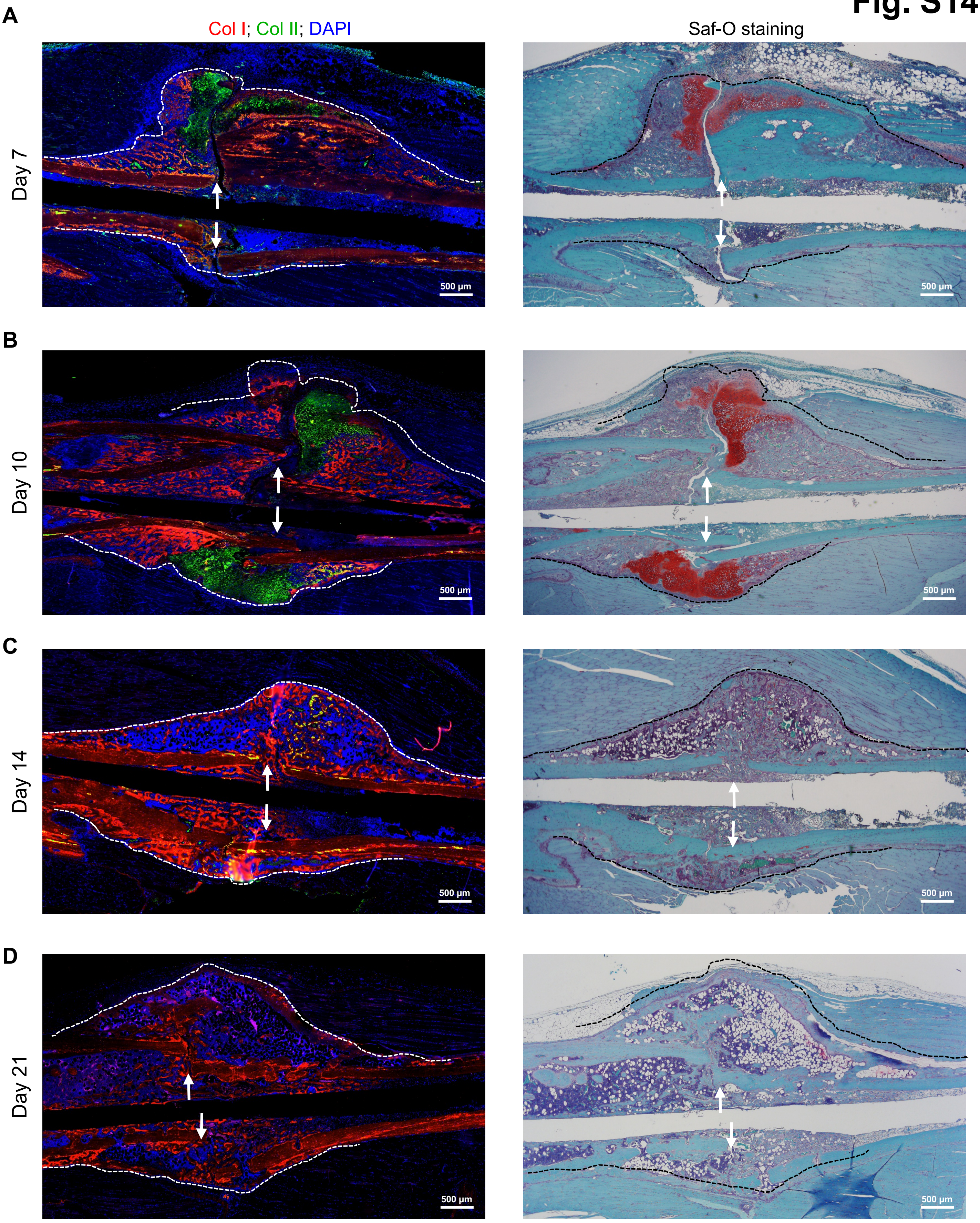
A
B
C
D
Col I; Col II; DAPI
Saf-O staining
Day 7
Day 10
Day 14
Day 21
500 μm

# Fig. S15

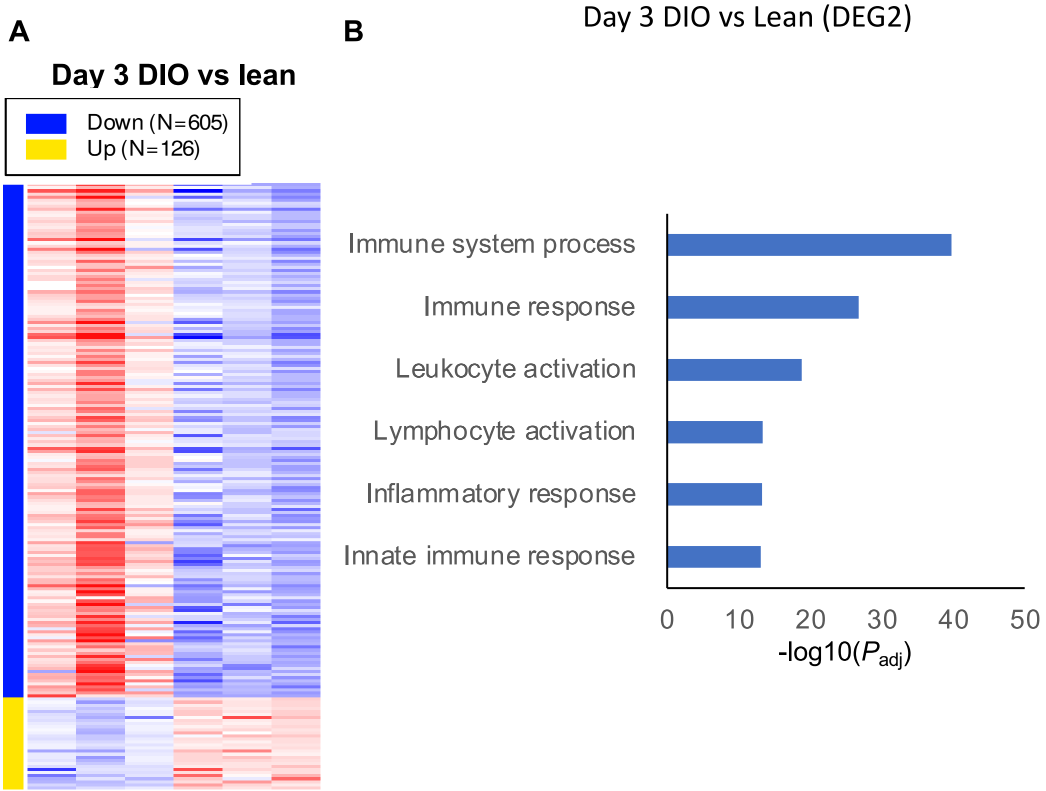
A
Day 3 DIO vs lean
Down (N=605)
Up (N=126)
B
Day 3 DIO vs Lean (DEG2)
Immune system process
Immune response
Leukocyte activation
Lymphocyte activation
Inflammatory response
Innate immune response
0
10
20
30
40
50
-log10($P_{adj}$)

# Fig. S16

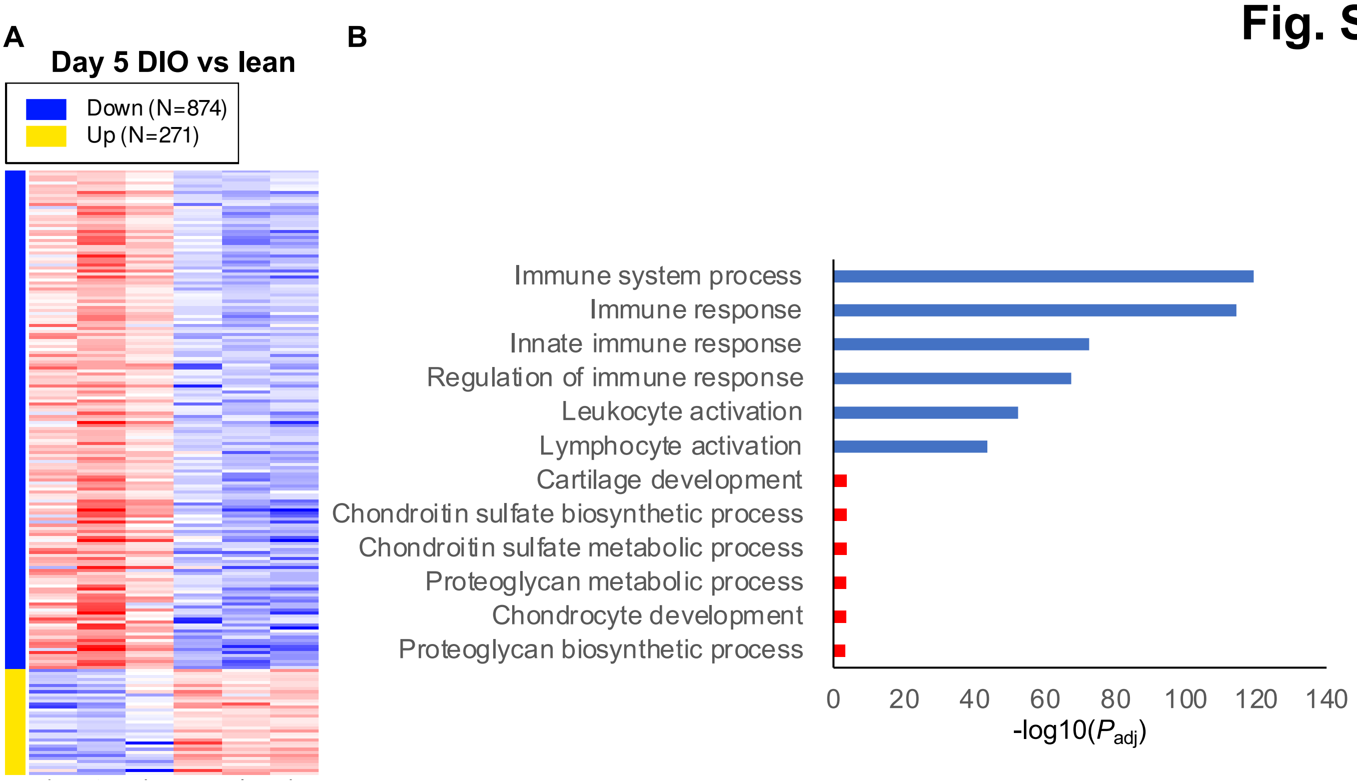
A
Day 5 DIO vs lean
Down (N=874)
Up (N=271)
B
Immune system process
Immune response
Innate immune response
Regulation of immune response
Leukocyte activation
Lymphocyte activation
Cartilage development
Chondroitin sulfate biosynthetic process
Chondroitin sulfate metabolic process
Proteoglycan metabolic process
Chondrocyte development
Proteoglycan biosynthetic process
0
20
40
60
80
100
120
140
-log10($P_{adj}$)

**Fig. S17**

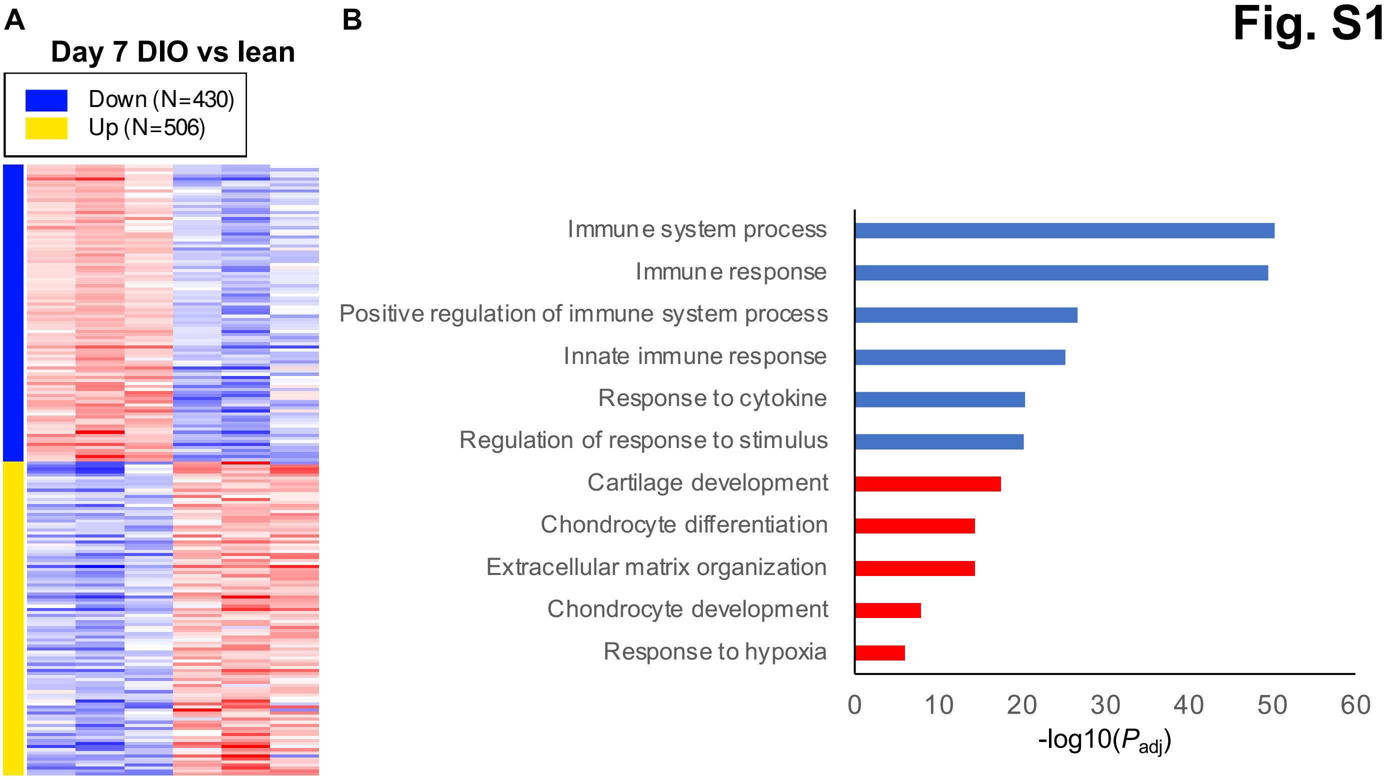
A
Day 7 DIO vs lean
Down (N=430)
Up (N=506)
B
Immune system process
Immune response
Positive regulation of immune system process
Innate immune response
Response to cytokine
Regulation of response to stimulus
Cartilage development
Chondrocyte differentiation
Extracellular matrix organization
Chondrocyte development
Response to hypoxia
0
10
20
30
40
50
60
-log10($P_{adj}$)

# Fig. S18

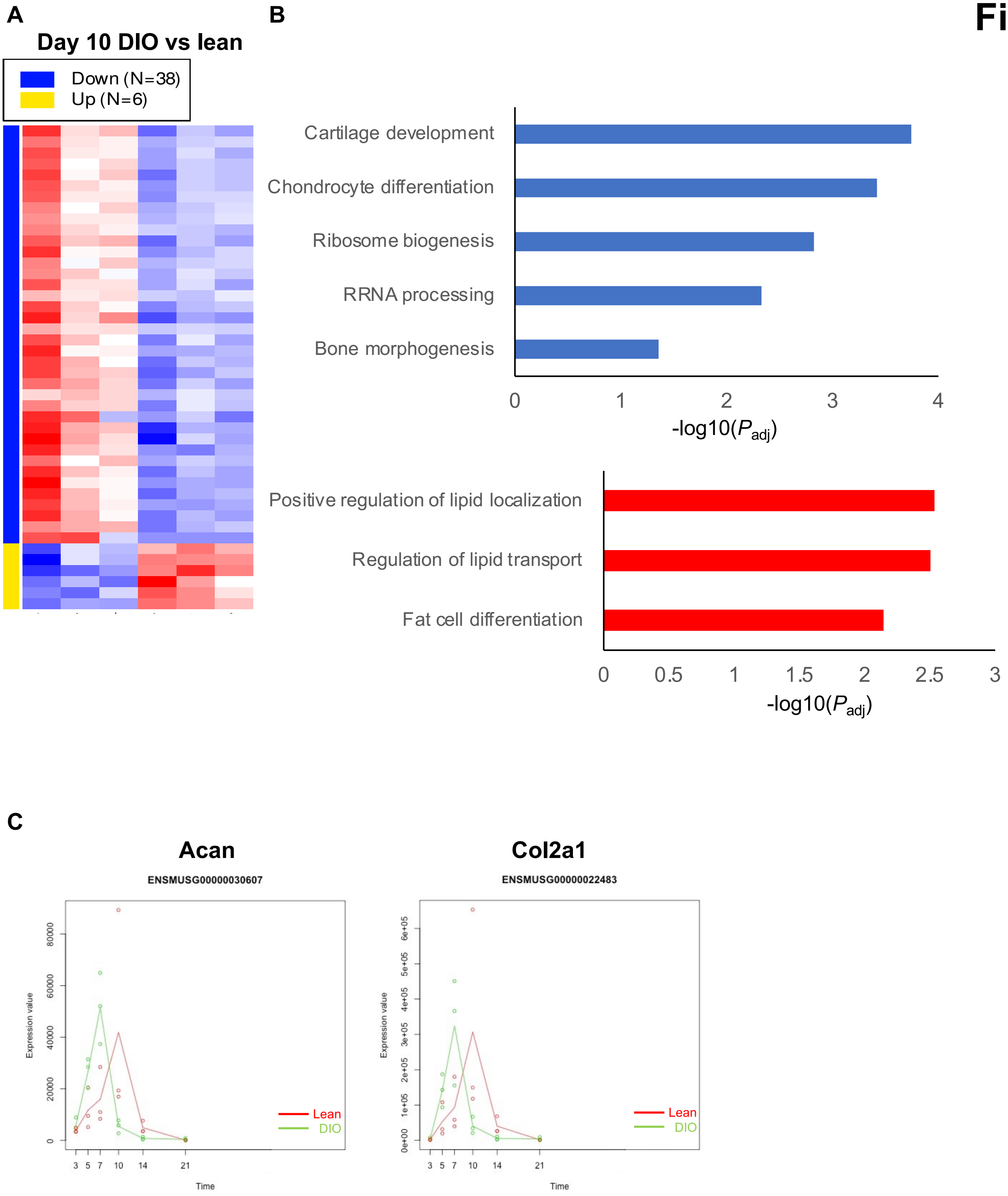
A
Day 10 DIO vs lean
Down (N=38)
Up (N=6)
B
Cartilage development
Chondrocyte differentiation
Ribosome biogenesis
RRNA processing
Bone morphogenesis
0
1
2
3
4
-log10($P_{adj}$)
Positive regulation of lipid localization
Regulation of lipid transport
Fat cell differentiation
0
0.5
1
1.5
2
2.5
3
-log10($P_{adj}$)
C
Acan
ENSMUSG00000030607
Expression value
0
20000
40000
60000
80000
3 5 7 10 14 21
Time
Lean
DIO
Col2a1
ENSMUSG00000022483
Expression value
0e+00
1e+05
2e+05
3e+05
4e+05
5e+05
6e+05
3 5 7 10 14 21
Time
Lean
DIO

Fig. S19

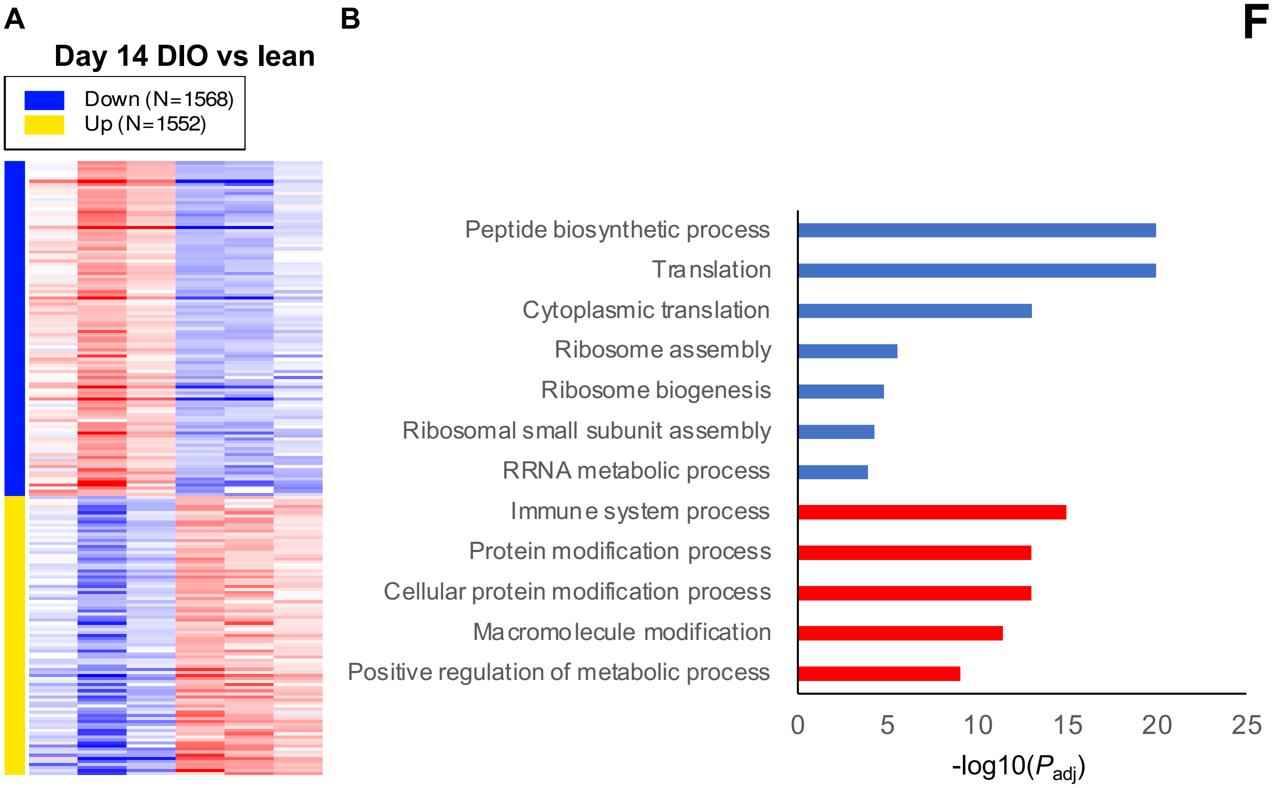
A
Day 14 DIO vs lean
Down (N=1568)
Up (N=1552)
B
Peptide biosynthetic process
Translation
Cytoplasmic translation
Ribosome assembly
Ribosome biogenesis
Ribosomal small subunit assembly
RRNA metabolic process
Immune system process
Protein modification process
Cellular protein modification process
Macromolecule modification
Positive regulation of metabolic process
0
5
10
15
20
25
-log10(Padj)

# Fig. S20

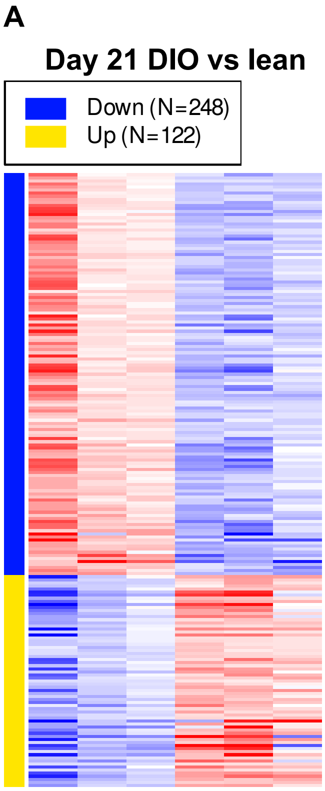
A
Day 21 DIO vs lean
Down (N=248)
Up (N=122)

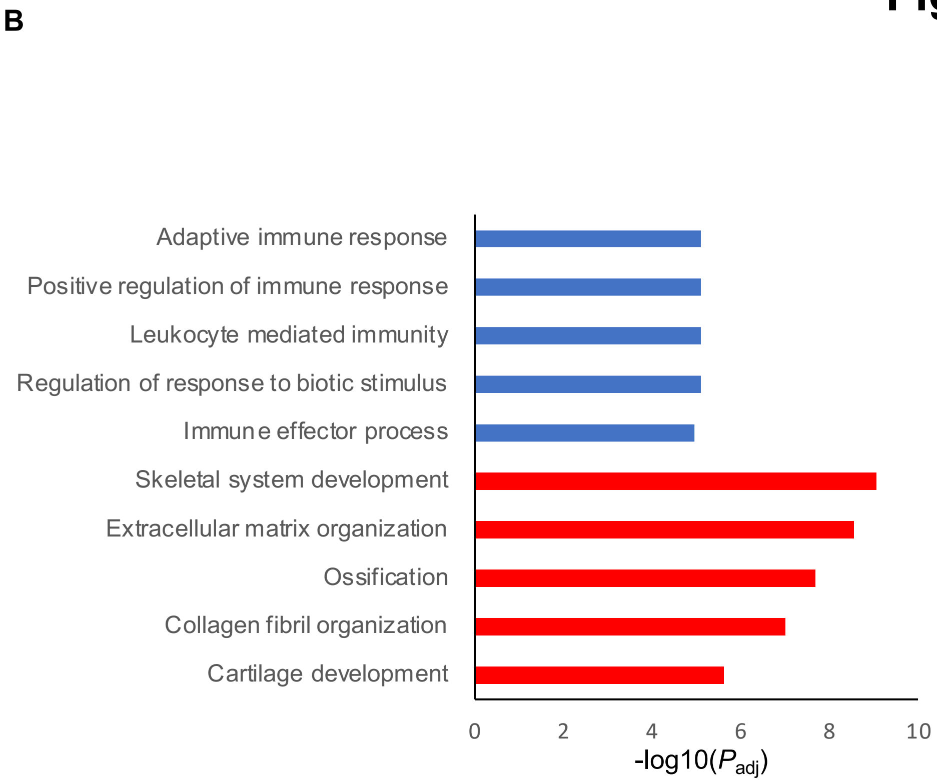
B
Adaptive immune response
Positive regulation of immune response
Leukocyte mediated immunity
Regulation of response to biotic stimulus
Immune effector process
Skeletal system development
Extracellular matrix organization
Ossification
Collagen fibril organization
Cartilage development
0
2
4
6
8
10
-log10(Padj)

Fig. S21

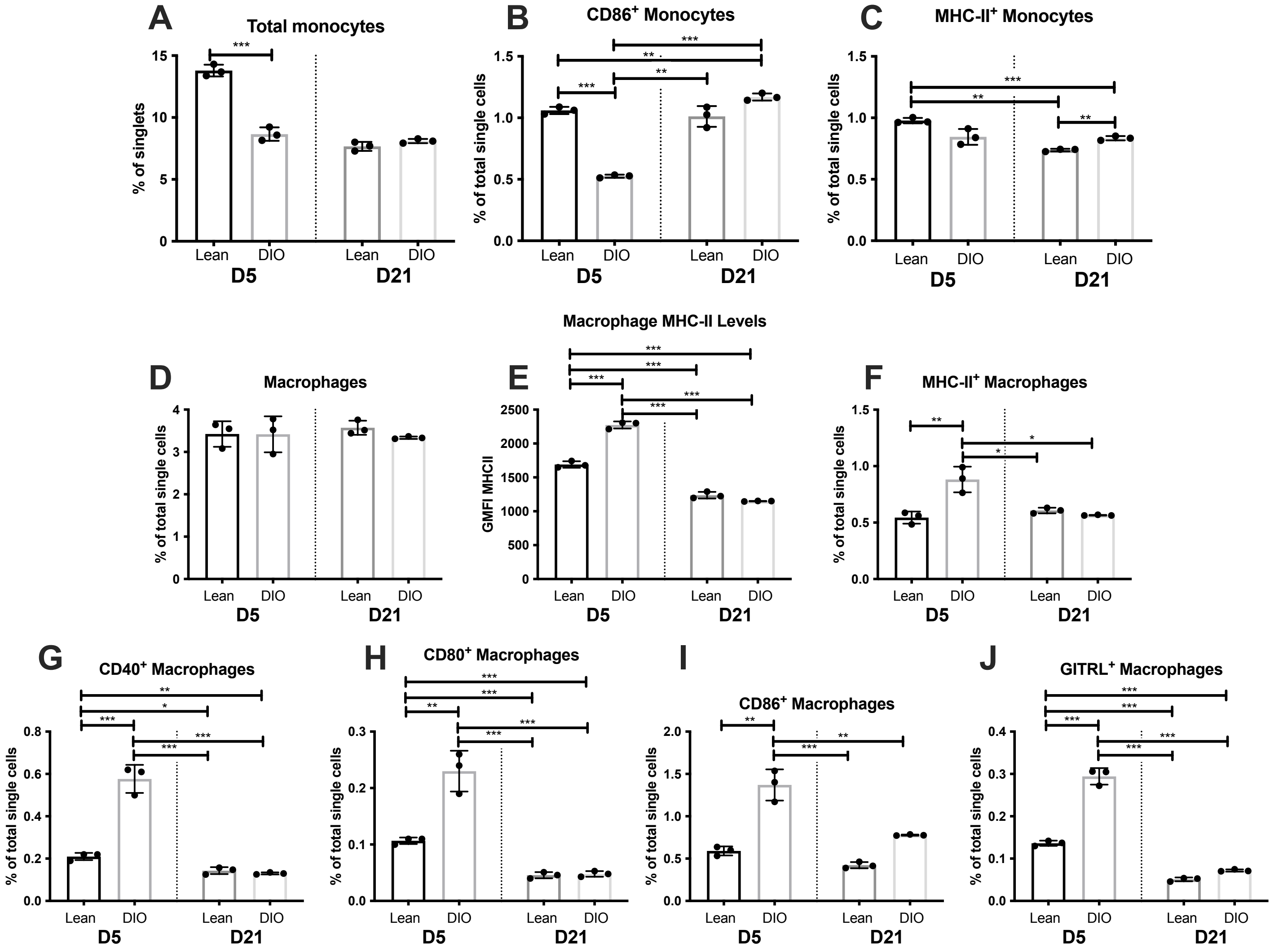
A
Total monocytes
% of singlets
Lean
DIO
D5
D21
B
CD86+ Monocytes
% of total single cells
C
MHC-II+ Monocytes
Macrophage MHC-II Levels
D
Macrophages
E
GMFI MHCII
F
MHC-II+ Macrophages
G
CD40+ Macrophages
H
CD80+ Macrophages
I
CD86+ Macrophages
J
GITRL+ Macrophages

# Fig. S22

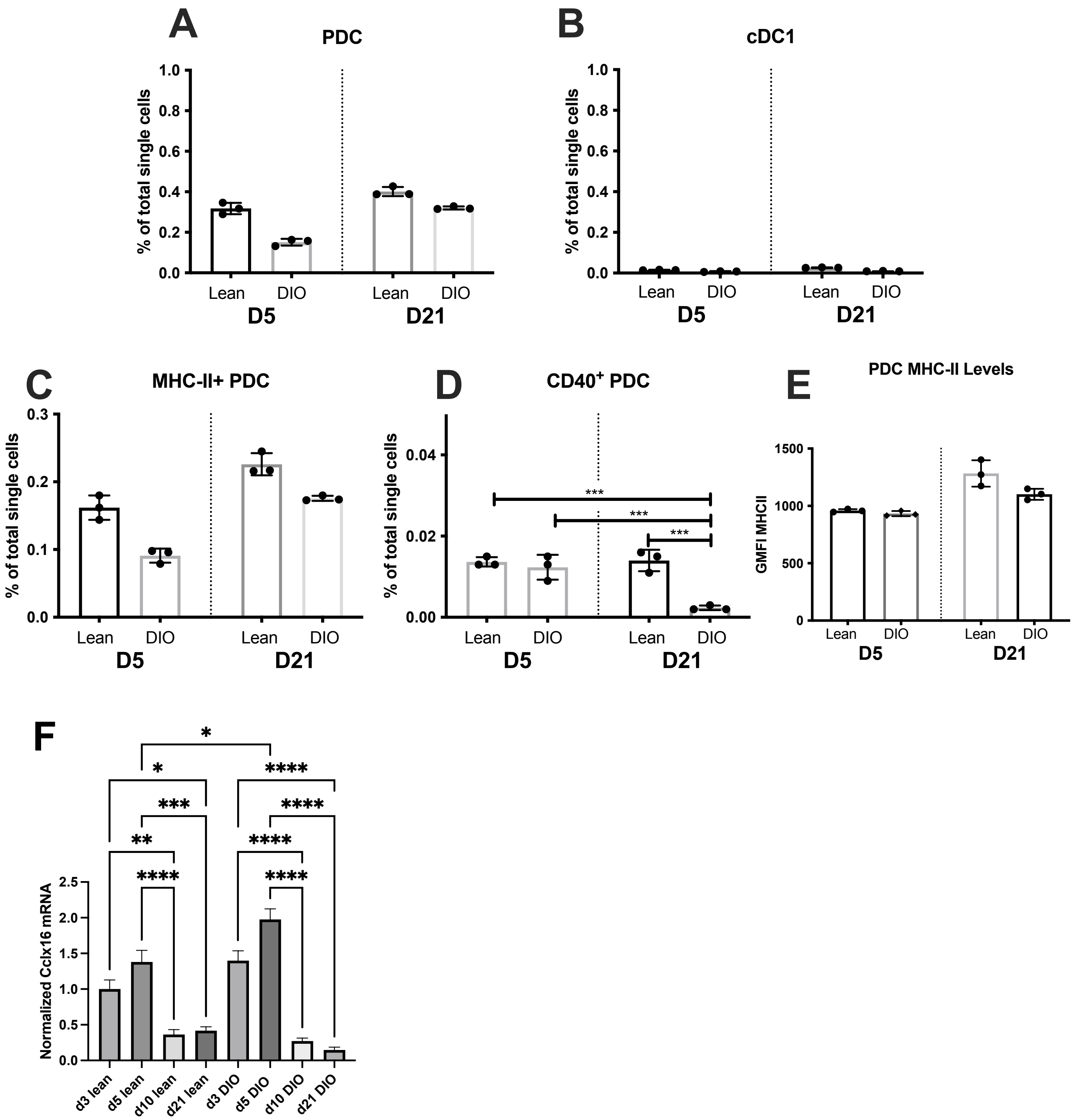

A
PDC
% of total single cells
Lean
DIO
D5
D21
B
cDC1
C
MHC-II+ PDC
D
CD40+ PDC
***
E
PDC MHC-II Levels
GMFI MHCII
F
Normalized Cclx16 mRNA
*
**
***
****
d3 lean
d5 lean
d10 lean
d21 lean
d3 DIO
d5 DIO
d10 DIO
d21 DIO

# Fig. S23

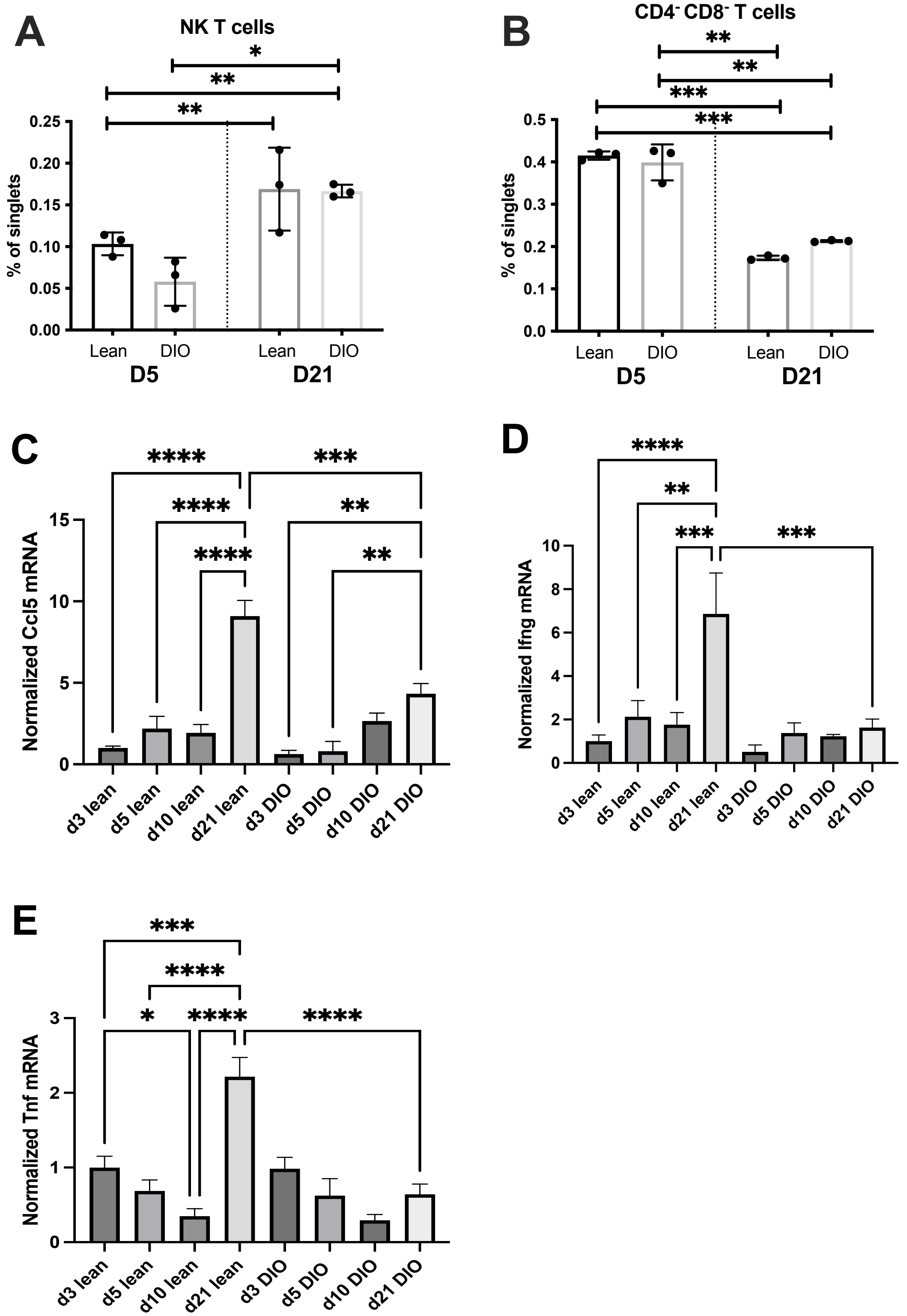
A
NK T cells
% of singlets
0.25
0.20
0.15
0.10
0.05
0.00
Lean
DIO
Lean
DIO
D5
D21
*
**
**
B
CD4⁻ CD8⁻ T cells
% of singlets
0.5
0.4
0.3
0.2
0.1
0.0
Lean
DIO
Lean
DIO
D5
D21
**
**
***
***
C
Normalized Ccl5 mRNA
15
10
5
0
d3 lean
d5 lean
d10 lean
d21 lean
d3 DIO
d5 DIO
d10 DIO
d21 DIO
****
****
****
***
**
**
D
Normalized Ifng mRNA
10
8
6
4
2
0
d3 lean
d5 lean
d10 lean
d21 lean
d3 DIO
d5 DIO
d10 DIO
d21 DIO
****
**
***
***
E
Normalized Tnf mRNA
3
2
1
0
d3 lean
d5 lean
d10 lean
d21 lean
d3 DIO
d5 DIO
d10 DIO
d21 DIO
***
****
*
****
****

# Fig. S24

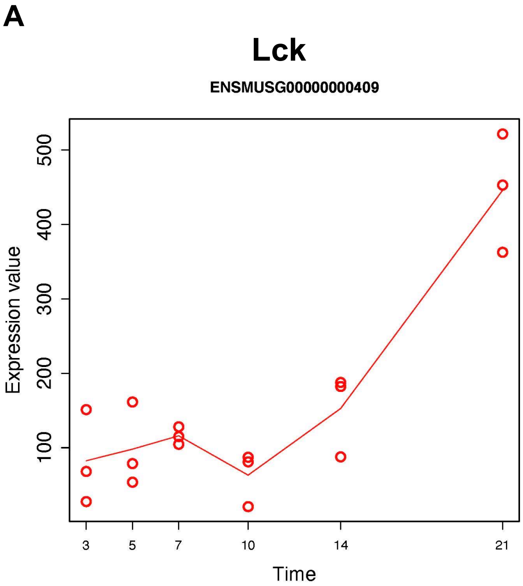
A
Lck
ENSMUSG00000000409
Expression value
100
200
300
400
500
3
5
7
10
14
21
Time

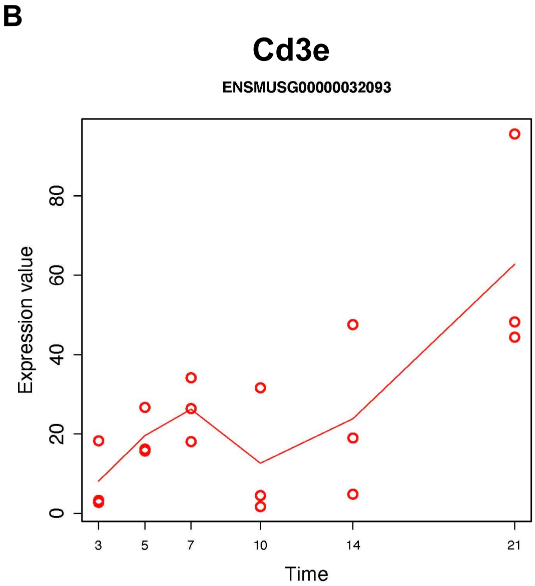
B
Cd3e
ENSMUSG00000032093
Expression value
0
20
40
60
80
3
5
7
10
14
21
Time

# Fig. S25

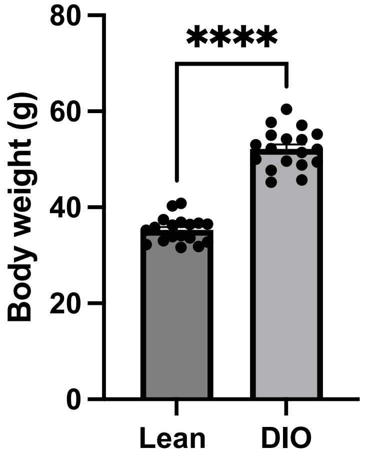

Supplement: Supplementary Figure 1 — (A-D, left) IF co-staining of Col I (red) and Col II (green). DAPI stains nuclei (blue). White arrows in each panel point to the fracture line. The dashed white line outlines the callus. The images show the full callus area from which panels in Figures 1A-D were cropped. The scale bar = 500 µm. (A-D, right) Safranin O/fast green (Saf-O) staining of the same sections shown in the left panels. The reddish orange staining denotes the extracellular matrix of the soft callus. The scale bar = 500 µm. [file DataSheet_1.pdf]
